# Exercise-induced GPLD1 is associated with neuroprotection and improvement of hippocampal dysfunction in an Alzheimer’s disease model

**DOI:** 10.1007/s11011-026-01857-1

**Published:** 2026-04-21

**Authors:** Muaz Belviranlı, Nilsel Okudan, Tuğba Sezer

**Affiliations:** School of Medicine, Department of Physiology, Selçuk University, Konya, 42131 Turkey

**Keywords:** GPLD1, Alzheimer's disease, Exercise, Hippocampal signaling, Exerkines

## Abstract

This study investigated the therapeutic potential of exercise training and the associated role of Glycosylphosphatidylinositol-specific phospholipase D1 (GPLD1) in an experimental Alzheimer’s disease (AD) model. Using a D-galactose/AlCl_3_ induced rodent model, we evaluated behavioral impairments, AD pathological markers, and the expression of key signaling pathways linked to neurodegeneration and neuroprotection, alongside and neurotrophic factors. Male rats were divided into Control, AD, Exercise, and AD + Exercise groups. The AD model exhibited significant deficits in locomotor activity, exploratory behavior, and spatial learning/memory, alongside elevated hippocampal Aβ_1–42_, tau protein, AChE activity. This was accompanied by overactivation of pathways implicated in neuroinflammation and pathology (NF-κB, mTOR, GSK-3β), and downregulation of pathways crucial for neuronal survival and plasticity (Wnt3a/β-catenin, PI3K/Akt), in addition to reduced GPLD1 and BDNF. Exercise intervention significantly ameliorated these behavioral deficits, increased hippocampal BDNF, GPLD1, and restored activity in pro-survival pathways (Wnt3a/β-catenin, PI3K/Akt) while reducing AD pathology markers. Notably, the AD + Exercise group demonstrated substantial recovery in most parameters compared to the AD group. Furthermore, plasma and liver GPLD1 levels were markedly upregulated by exercise. These findings collectively suggest that exercise training is associated with robust neuroprotection, correlating with systemic GPLD1 upregulation, and highlights the GPLD1 axis for future therapeutic investigation in AD.

**Graphical abstract:**

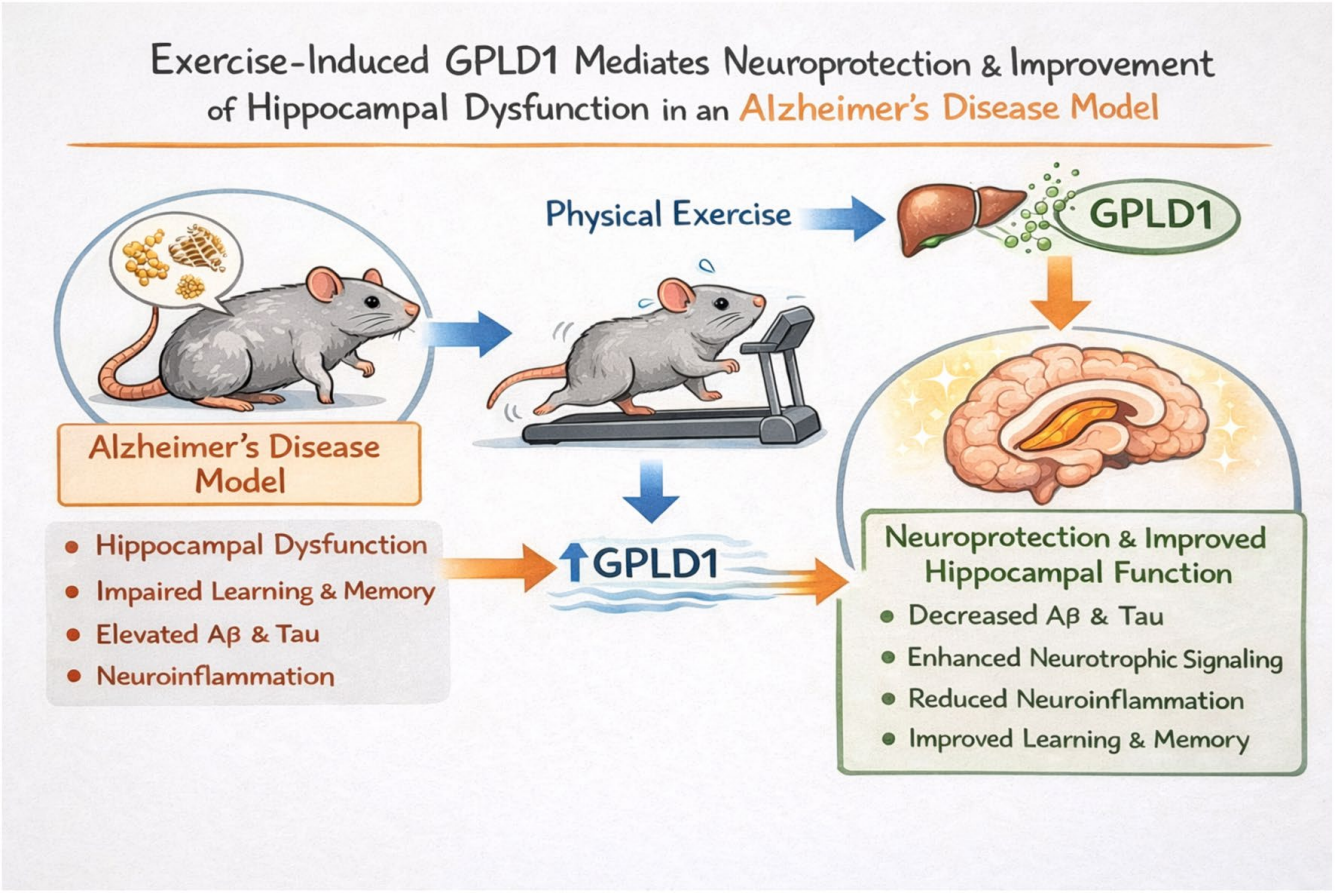

## Introduction

Alzheimer’s disease (AD) is the most prevalent form of dementia, affecting an estimated 60–80% of all cases and presenting a critical global health challenge ((2023) ). Its hallmark neuropathologies include the accumulation of extracellular amyloid-beta (Aβ) plaques and intracellular hyperphosphorylated tau neurofibrillary tangles (NFTs), synergistically driving profound synaptic loss and cognitive impairment (Dubois et al. 2021, Long and Holtzman 2019). The precise mechanisms underpinning AD’s multifactorial etiology remain largely elusive, yet beyond Aβ and tau, factors such as cholinergic deficits, neuroinflammation, and mitochondrial dysfunction are increasingly implicated (Zhang et al. 2024). The hippocampus, vital for learning and memory, is particularly vulnerable to these insults, exhibiting reduced neurogenesis and impaired neural morphology (Kamatham et al. 2024, Mufson et al. 2015, Geigenmüller et al. 2024). Consequently, various experimental models of AD—ranging from amyloid-beta infusion and transgenic rodent models to chemically induced models using D-galactose and AlCl_3_—consistently demonstrate cognitive and non-cognitive deficits, including severe learning and memory impairments, decreased locomotor activity, and increased anxiety (Hoveida et al. 2011, Dao et al. 2014, Belviranlı and Okudan 2024).

To understand how these behavioral outcomes arise, examining dysregulated molecular pathways is crucial. Neurotrophic factors, particularly brain-derived neurotrophic factor (BDNF), offer neuroprotection against Aβ-induced neurodegeneration, maintaining neuronal viability and mitigating synaptic anomalies (Numakawa et al. 2018, Pins et al. 2019, Mitroshina et al. 2020). In AD, key pro-survival pathways like wingless-related integration site 3a (Wnt3a)/β-catenin and phosphatidylinositol 3-kinase (PI3K)/protein kinase B (Akt) are suppressed, while nuclear factor kappa B (NF-κB) and mammalian target of rapamycin (mTOR) hyperactivation are linked to neuroinflammation and autophagy defects (Zhang et al. 2024, Kang and Cho 2015, Koo et al. 2017, Jia et al. 2019). Additionally, overactive glycogen synthase kinase 3β (GSK-3β) plays a central role by promoting both amyloid precursor protein (APP) amyloidogenic processing and tau hyperphosphorylation (Zhang et al. 2024, Llorens-Martín et al. 2014).

The absence of an effective pharmacological AD treatment underscores the need for alternative strategies. Physical exercise has emerged as a promising non-pharmacological intervention, with observational studies suggesting its role in both prevention and treatment (Sujkowski et al. 2022, Iso-Markku et al. 2022, Sá Leitão et al. 2025, Dou et al. 2025). In AD, its neurobeneficial effects are pronounced in the hippocampus, where it stimulates neurogenesis, enhances synaptic plasticity, and improves memory (Huuha et al. 2022, Hao et al. 2023). Numerous rodent studies indicate that regular exercise training reduces Aβ and tau accumulation, confers neuroprotection (Lu et al. 2017), attenuates inflammation (Lu et al. 2017), and improves both cognitive (Numakawa et al. 2018, Cho et al. 2015, Maliszewska-Cyna et al. 2016) and non-cognitive functions (Dao et al. 2014, Belviranlı and Okudan 2019). The exercise-induced reduction in Aβ and tau levels is linked to the activation of Aβ-degrading enzymes (Koo et al. 2017, Moore et al. 2016), enhanced synaptic plasticity (Jiang et al. 2015), increased neurogenesis (Jiang et al. 2015, Ke et al. 2011) and upregulation of neurotrophic factors (Belviranlı and Okudan 2019, Nigam et al. 2017). Importantly, exercise counteracts the molecular imbalances seen in AD by restoring Wnt3a/β-catenin and PI3K/ Akt activity, and suppressing the pathological overactivation of mTOR, NF-κB, and GSK-3β (Kang and Cho 2015, Jia et al. 2019, Llorens-Martín et al. 2014, Hao et al. 2023).

While physical activity clearly benefits brain health, its cellular and molecular underpinnings remain largely unclear. Growing evidence suggests that systemic changes, involving circulating factors called “exerkines” released during exercise, mediate the brain’s responses. This concept is supported by parabiosis experiments, which demonstrate that the infusion of young blood can reverse age-related cognitive decline (Villeda et al. 2011, Katsimpardi et al. 2014, Horowitz and Villeda 2017). Among these exerkines, Glycosylphosphatidylinositol-specific phospholipase D1 (GPLD1), an enzyme predominantly expressed in the liver that cleaves GPI-anchored proteins, is a compelling candidate (Raymond et al. 1994, Mann et al. 2004, Fagerberg et al. 2014). A seminal study showed liver-derived GPLD1 is induced by regular exercise in mice and is associated with exercise-induced cognitive improvements in both aged and young animals (Horowitz et al. 2020). Physically active older adults exhibit higher plasma GPLD1 (Marlatt et al. 2025), and mere GPLD1 overexpression in the liver in sedentary mice mimicked exercise benefits, increasing hippocampal BDNF, neurogenesis, and cognitive function (Horowitz et al. 2020). Mechanistically, GPLD1 overexpression stimulates galectin-3-binding protein (GAL3BP) secretion, inhibiting APP processing and Aβ production (Seki et al. 2020). Reduced glycosylphosphatidylinositol-specific phospholipase D (GPI-PLD) expression has been observed in neurodegenerative prion diseases, further implicating GPLD1 (Jin et al. 2015). Despite these indicators, a direct investigation into the role of both hippocampal and liver-derived GPLD1 in AD pathology is currently absent.

While previous studies, such as Horowitz et al. (Horowitz et al. 2020), have established the role of GPLD1 in age-related cognitive decline, its specific involvement and multi-tissue expression profile in the context of AD pathology remain unexplored. Therefore, the novelty of this study lies in extending the GPLD1 paradigm to a chemical AD model, simultaneously evaluating its expression across the liver-blood-brain axis to determine if this mechanism is preserved or altered under neurodegenerative conditions.

This study aims to evaluate and characterize a D-galactose/AlCl_3_-induced experimental AD model, focusing on learning and memory functions, and critically, to explore alterations in central (hippocampal) and peripheral (plasma/liver) GPLD1 levels. The study will also elucidate regular exercise’s impact on AD progression, cognitive/non-cognitive functions, and relevant molecular pathways (Aβ_1−42_, tau, acetylcholinesterase (AChE) activity, mRNA expression of Wnt3a/β-catenin, PI3K/Akt, mTOR, NF-κB and GSK-3β), hypothesizing their linkage to GPLD1-associated neuroprotection. By integrating these systemic, molecular, and behavioral analyses, this research seeks novel insights into how exercise confers neuroprotective benefits in AD, potentially identifying the GPLD1 axis as a subject for future therapeutic investigation.

## Methods

### Experimental animals and ethical considerations

The study involved forty-eight male Wistar rats, aged four months and with a body weight ranging from 300 to 400 g. The sample size was determined a priori using G*Power software (v3.1). Based on previous similar behavioral studies, a minimum of 10 animals per group was calculated to achieve 80% power at an alpha level of 0.05. To account for potential attrition, *n* = 12 was selected. Male rats were exclusively used to avoid the confounding neuroprotective fluctuations of the estrous cycle, aligning with standard initial exploratory models in neurodegeneration. The rats were maintained in a controlled vivarium with a 12-hour light-dark cycle, a stable ambient temperature of 22 ± 2 °C, and 50% relative humidity. They were group-housed in polycarbonate cages with free access to standard pellet diet and water.

This study was reported in accordance with the Animal Research: Reporting of in Vivo Experiments (ARRIVE) guidelines. The experimental design and all associated procedures were prospectively reviewed and approved by the Local Ethics Committee on Animal Experiments at the Selçuk University (Decision No: 2024-9).

### Experimental design and animal groups

**Fig. 1:**
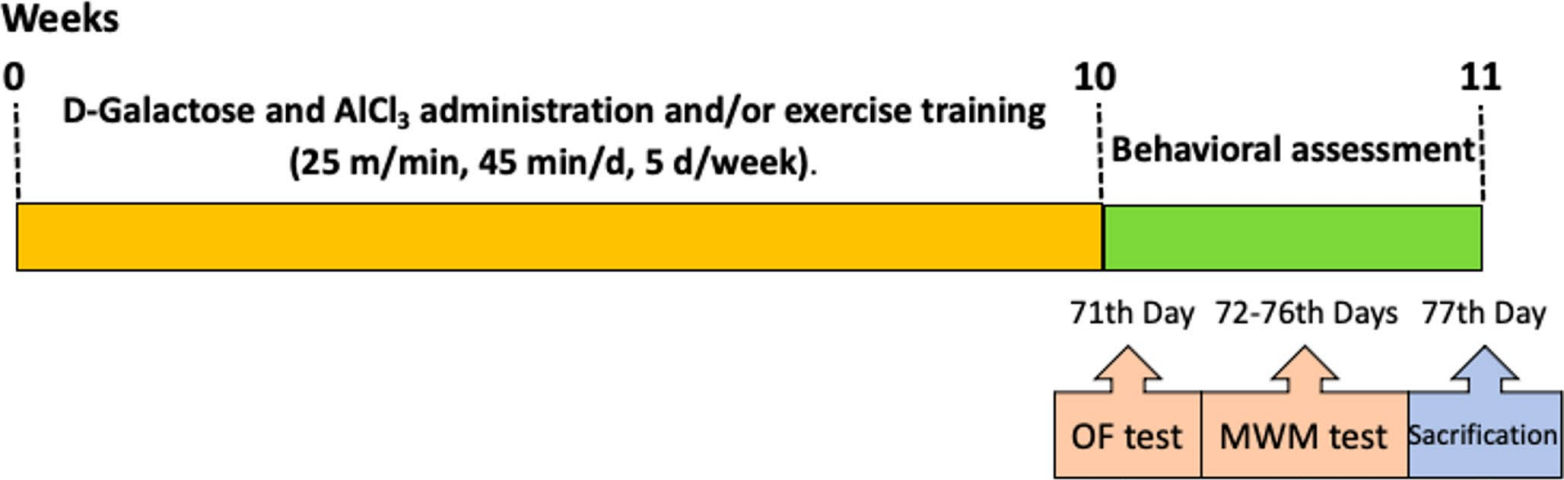
Experimental design flowchart

Forty-eight rats were randomly assigned into four experimental groups (*n* = 12/group) using a simple randomization procedure: Control (C), AD (A), Exercise (E), and AD + Exercise (AE). A comprehensive overview of the experimental workflow is provided in Fig. 1. Animals in the C group received only saline as a vehicle and did not undergo any other intervention. For 10 weeks, the A and AE groups received daily administrations of D-galactose (60 mg kg^− 1^) and AlCl_3_ (200 mg kg^− 1^) to model AD pathology. During this same period, the E and AE groups participated in a regular exercise training protocol performed five days per week. Twenty-four hours after the final exercise session, behavioral parameters were evaluated using the Open Field (OF), and Morris Water Maze (MWM) tests. Subsequently, 24 h after the completion of behavioral assessments, blood samples were collected under anesthesia. Immediately thereafter, euthanasia was performed via cervical dislocation, and samples of the hippocampus and liver were rapidly dissected and procured for subsequent analyses.

### Induction of the experimental model of Alzheimer’s disease

To establish the experimental AD model, rats within the A and AE groups underwent a daily co-administration regimen for a period of 10 weeks. This protocol involved the intraperitoneal injection of 60 mg kg^− 1^ D-galactose (Sigma Aldrich, Cat. No: G0625), dissolved in physiological saline, alongside the intragastric gavage of 200 mg kg^− 1^ AlCl_3_ (Sigma Aldrich, Cat. No: 294713), prepared in distilled water. This specific combination, utilized for AD induction, has been previously validated and shown to be effective in replicating key aspects of the disease (Chiroma et al. 2018).

Various animal models are employed to investigate the diverse facets of AD. Since familial AD (FAD) accounts for less than 1% of all AD cases, commonly used experimental models relying on transgenic animals are not considered ideal for studying sporadic AD (SAD) (LaFerla and Green 2012). While intracerebroventricular streptozotocin (STZ) injection is a widely recognized and robust model for SAD (León-Arcia et al. 2026)—primarily simulating brain insulin resistance—it requires invasive stereotaxic cranial surgery. In contrast, systemic chemical induction methods, involving agents such as D-galactose and AlCl_3_, provide another highly suitable, non-surgical alternative to model SAD (Dam and Deyn 2006). Within experimental paradigms, D-galactose is commonly used to accelerate the aging process, as it reliably induces behavioral and neurobiological changes that closely mimic those of natural senescence (Duan et al. 2018). AlCl_3_, on the other hand, contributes to neurodegeneration by impairing axonal transport and synaptic integrity, leading to the degeneration of cholinergic neurons and subsequent learning impairments (Chiroma et al. 2018, Alghamdi 2018). Critically, the experimental AD model established through the combined application of D-galactose and AlCl_3_ has been reported to better simulate both the cognitive dysfunctions and pathological alterations characteristic of clinically diagnosed AD individuals (Xiao et al. 2011). In light of these advantages, the D-galactose and AlCl_3_ combination was specifically chosen in the present study to induce a robust SAD model. Conversely, the C and E groups, which were not subjected to AD induction, received only the corresponding vehicle solutions—physiological saline intraperitoneally and distilled water intragastrically.

### Exercise intervention protocol

All physical exercise protocols were conducted using a motorized rodent treadmill (MAY-TME 0804, Commat, Ankara, Turkey). To provide a motivational stimulus for the animals, an electric shock grid was positioned at the rear barrier, while diurnal variations were minimized by performing all exercise sessions at the same time daily. Following a 1-week acclimatization period, rats assigned to the exercise groups (E and AE) underwent a 10-week exercise regimen, which closely resembled protocols utilized in our previous studies (Belviranlı et al. 2012). This regimen consisted of 45-minute daily treadmill runs, five days per week, at a constant speed of 25 m min^− 1^. This specific exercise intensity has been previously validated in Wistar rats to correspond to approximately 55–60% of the maximal oxygen consumption (VO_2max_) in rats (Bedford et al. 1979). To minimize stress as a confounding factor, the use of the electric shock grid was kept to an absolute minimum.

Before starting the training protocol, a 1-week adaptation protocol was implemented, which included a gradual increase in both speed and duration. To account for potential handling and treadmill-associated stress, animals in the non-exercise groups (C and A) were habituated to the treadmill environment twice per week for 5 min at a low speed of 10 m min^− 1^.

### Behavioral assessment

Upon the completion of the 10-week experimental period, all study groups were subjected to a battery of behavioral tests, commencing with the OF test, followed by the MWM test. All behavioral evaluations were rigorously conducted in a dedicated, sound- and light-isolated room, under consistent environmental conditions, and performed by a single experimenter blinded to the experimental group allocations. Utmost care was taken to minimize any extraneous factors that might influence the animals’ emotional state. The testing room was equipped with adequate ventilation. To prevent inter-animal cueing from olfactory or other residues, the OF apparatus was thoroughly cleaned with 70% ethanol, ventilated and allowed to dry completely following each rat’s trial. All behavioral sessions were consistently conducted at the same time each day to control for diurnal variations. Data acquisition was performed online via a digital camera system, and the recorded videos were subsequently analyzed offline using specialized analytical software (EthoVision XT 10.0, Noldus Information Technology, Wageningen, Netherlands). Inter-rater reliability was confirmed on a subset of videos. Exclusion criteria were predefined; for instance, rats exhibiting floating behavior (> 20% of the trial time in MWM) without searching were to be excluded, though no animals met this criterion. The specific protocols for all behavioral assessments largely adhered to methodologies established in our prior researches (Belviranlı and Okudan 2024, Belviranlı and Okudan 2019, Belviranlı et al. 2013, Belviranlı and Okudan 2018).

#### Open field test

Spontaneous locomotor activity, exploratory behavior and anxiety level in rats were evaluated using a square, black, wooden OF apparatus (80 cm x 80 cm x 40 cm height). The wooden surface of the OF apparatus was sealed with a non-porous coating to prevent the absorption of urine odors or other conspecific cues. Each rat was individually introduced to the center of the arena and permitted free movement for a 5-minute observation period. The arena was conceptually divided into a central zone and a peripheral zone for subsequent data analysis. Parameters meticulously recorded included: total distance traveled, duration spent within the central zone, frequency of rearing episodes, instances of defecation and grooming, average velocity during the trial, total time exhibiting mobile behavior, and the number of transitions between the peripheral and central regions.

#### Morris water maze test

The MWM test is a widely recognized and extensively used behavioral paradigm for assessing spatial learning and memory functions in rodents. The MWM apparatus was a circular tank (150 cm in diameter × 60 cm deep) filled with water maintained at 25 ± 1 °C. The MWM paradigm involved two fundamental tasks: a training (acquisition) task and a subsequent probe trial.

##### Training task (Acquisition)

During the initial training phase, rats learned to locate a hidden platform. The tank was conceptually delineated into four equal quadrants (northeast, northwest, southeast, and southwest). A submerged, square escape platform (10 × 10 cm) was fixed in the southeast quadrant, approximately 2 cm below the water surface, and its position remained constant throughout the training trials. The water in the tank was rendered opaque using non-toxic, water-based black dye to effectively obscure the platform. Various distinct extra-maze visual cues were strategically placed around the tank’s perimeter to facilitate the rats’ spatial orientation. Rats performed a total of 16 trials, organized into four blocks of 4 trials daily over four consecutive days, with the daily performance averaged. For each training day, four different starting positions were randomized. For each individual trial, animals were allowed a maximum of 60 s to locate the platform. If a rat failed to find the platform within 60 s, the experimenter gently guided it to the platform and allowed it to remain there for 30 s. Recorded metrics during this acquisition phase included: escape latency (time to reach the platform), total distance traversed, and average swimming speed.

##### Probe test

A probe test, evaluating memory retention, was conducted 24 h after the final training trial, following the removal of the hidden platform. During this 90-second test, rats were permitted to swim freely in the pool. The parameters assessed for memory consolidation included: cumulative time spent in the target quadrant and the number of crossings over the virtual platform location.

### Sample collection and tissue preparation

Twenty-four hours following the final behavioral assessment, blood samples were collected from the hearts of rats under ketamine HCl/xylazine (60/10 mg kg^− 1^) anesthesia. Subsequent to blood collection, animals were humanely euthanized by cervical dislocation. Immediately following euthanasia, target tissues, including the liver, and the hippocampus, were meticulously excised. Each tissue sample was promptly rinsed with ice-cold physiological saline to remove residual blood, then rapidly snap-frozen in liquid nitrogen and stored at -80 °C until further analysis. Concurrently, blood samples, collected directly into EDTA-containing tubes, were centrifuged at 1500 × g for 20 min immediately after collection to separate the plasma. The obtained plasma aliquots were also stored at -80 °C until biochemical assays were performed.

### Biochemical and molecular analyses

#### Biochemical analysis

Levels of GPLD1 in plasma, hippocampus, and liver tissues; Aβ_1−42_, tau protein, AChE, and BDNF in hippocampal tissue were quantitatively assessed using commercial ELISA kits in strict accordance with the manufacturers’ instructions. For the hippocampus and liver tissue analyses, samples were initially homogenized in ice-cold phosphate buffer (pH 7.4). The resulting homogenates were then centrifuged at 12,000 × g for 10 min at 4 °C to separate the supernatant, which was subsequently used for all ELISA determinations. Specifically, commercial kits were sourced to determine GPLD1 in plasma, hippocampus, and liver (Cat No: E1439Ra; BT LAB, Shanghai, China) and Aβ_1−42_ (Cat No: ELK4897; ELK Biotechnology, Wuhan, China), tau protein (Cat No: E1392Ra; BT LAB, Shanghai, China), AChE (Cat No: E-EL-R0355; ELabscience, Texas, USA), and BDNF (Cat No: ELK5459; ELK Biotechnology, Wuhan, China) in the hippocampus tissue. To ensure accurate protein quantification across all samples prior to ELISA, a commercial protein analysis kit was employed (Cat No: P011; ABP Bioscience, MD, USA). Each analysis was meticulously carried out according to the provided instructions from the respective manufacturers. The absorbance of the chromogenic reaction, reflecting the intensity of the yellow coloration, was read at the appropriate wavelength using a microplate reader (PowerWave XS, BioTek Instruments Inc., Winooski, USA). The concentrations of the relevant parameters in the experimental samples were subsequently calculated by correlating their optical density values with a standard curve, which was generated from the optical density values corresponding to known standard concentrations. To ensure rigorous reproducibility, all ELISA samples were run in duplicate. The intra-assay and inter-assay coefficients of variation (CV) were confirmed to be < 8% and < 10%, respectively, demonstrating high assay specificity and reliability.

#### Gene expression analysis

First, total RNA was isolated from hippocampal and liver tissue samples using PureZol RNA extraction reagent (Bio-Rad Laboratories, Hercules, CA, USA), strictly adhering to the manufacturer’s guidelines. The purity and concentration of the isolated RNA were subsequently determined by assessing the 260/280 absorbance ratio. Complementary DNA (cDNA) was then synthesized from the extracted RNA using the iScript cDNA Synthesis Kit (Cat. No: 1708891, Bio-Rad Laboratories, Hercules, CA, USA). Quantitative PCR reactions were performed in a 96-well format using the CFX Connect Real-Time PCR System (Bio-Rad Laboratories, Hercules, CA, USA) and SYBR Green Super Mix (Cat. No: 1708880, iQ SYBR Green Supermix, Bio-Rad Laboratories, Hercules, CA, USA), with target-specific primers (Table 1), following the manufacturer’s recommended thermal cycling conditions. Relative gene expression levels were quantified using the 2^⁻ΔΔCq^ method, normalizing target gene expression to the internal housekeeping control gene GAPDH, with the control group expression set to 1.0 (Schmittgen and Livak 2008). For RT-qPCR, primer efficiencies were validated using standard curves and fell within the acceptable range of 90–110%. The stability of the GAPDH housekeeping gene across all experimental and control groups was verified prior to normalization.

**Table 1:** Gene-specific primer sequences employed for RT-qPCR quantification

GPLD1	Forward	GTCGGAAAGTCATCACCAAGA
	Reverse	GTCCCGTTCTCCAGCATAAA
BDNF	Forward	TCATACTTCGGTTGCATGAAGG
	Reverse	ACACCTGGGTAGGCCAAGTT
mTOR	Forward	CGGGACTACAGAGAGAAGAAGA
	Reverse	CCAATTCAGCAGAGGGTCATAG
WNT3a	Forward	CATGAACCGTCACAACAATGAG
	Reverse	GGTGTTTCTCTACCACCATCTC
PI3K	Forward	GGCCTTGACTATATGGGATGTG
	Reverse	GAGAGTGTGCTGCTTGTTCT
AKT	Forward	AATGTGGGCTCATGGGTCTG
	Reverse	AGAGGGAGAGGGCCAGTTAG
GSK-3β	Forward	GAGTGGCGAGAAGAAAGATGAG
	Reverse	TGGCAGATCCCAAAGGAATG
Beta catenin	Forward	GATCCCATCCACGCAGTTT
	Reverse	CACCTGGTCCTCGTCATTTAG
GAPDH	Forward	ACTCCCATTCTTCCACCTTTG
	Reverse	CCCTGTTGCTGTAGCCATATT

### Statistical analysis

All statistical computations were performed utilizing SPSS 25.0 (IBM SPSS Inc.; Chicago, IL, USA) and GraphPad Prism 9.0 software (GraphPad Software Inc.; San Diego, CA, USA). Quantitative data are presented as the mean ± standard deviation (SD). The normality of data distribution for all variables was rigorously assessed using the Shapiro-Wilk test. For parameters measured in the OF test, MWM probe test, and all biochemical and molecular assays, data conforming to a normal distribution were analyzed using a one-way analysis of variance (ANOVA) followed by Tukey’s Honestly Significant Difference (HSD) test for multiple comparisons. Variables that did not exhibit a normal distribution were analyzed using the non-parametric Kruskal-Wallis test followed by Dunn’s test. For continuous numerical variables measured across different time points, such as the repeated measures obtained during the Morris Water Maze training task, repeated measures ANOVA was employed. In instances where repeated measures ANOVA indicated a significant difference among groups, Bonferroni-corrected Tukey’s test was utilized to pinpoint the specific groups responsible for the observed differences. A p-value less than 0.05 (*p* < 0.05) was considered to indicate statistical significance.

## Results

### Behavioral performance assessments

#### Locomotor activity and exploratory behavior

**Fig. 2:**
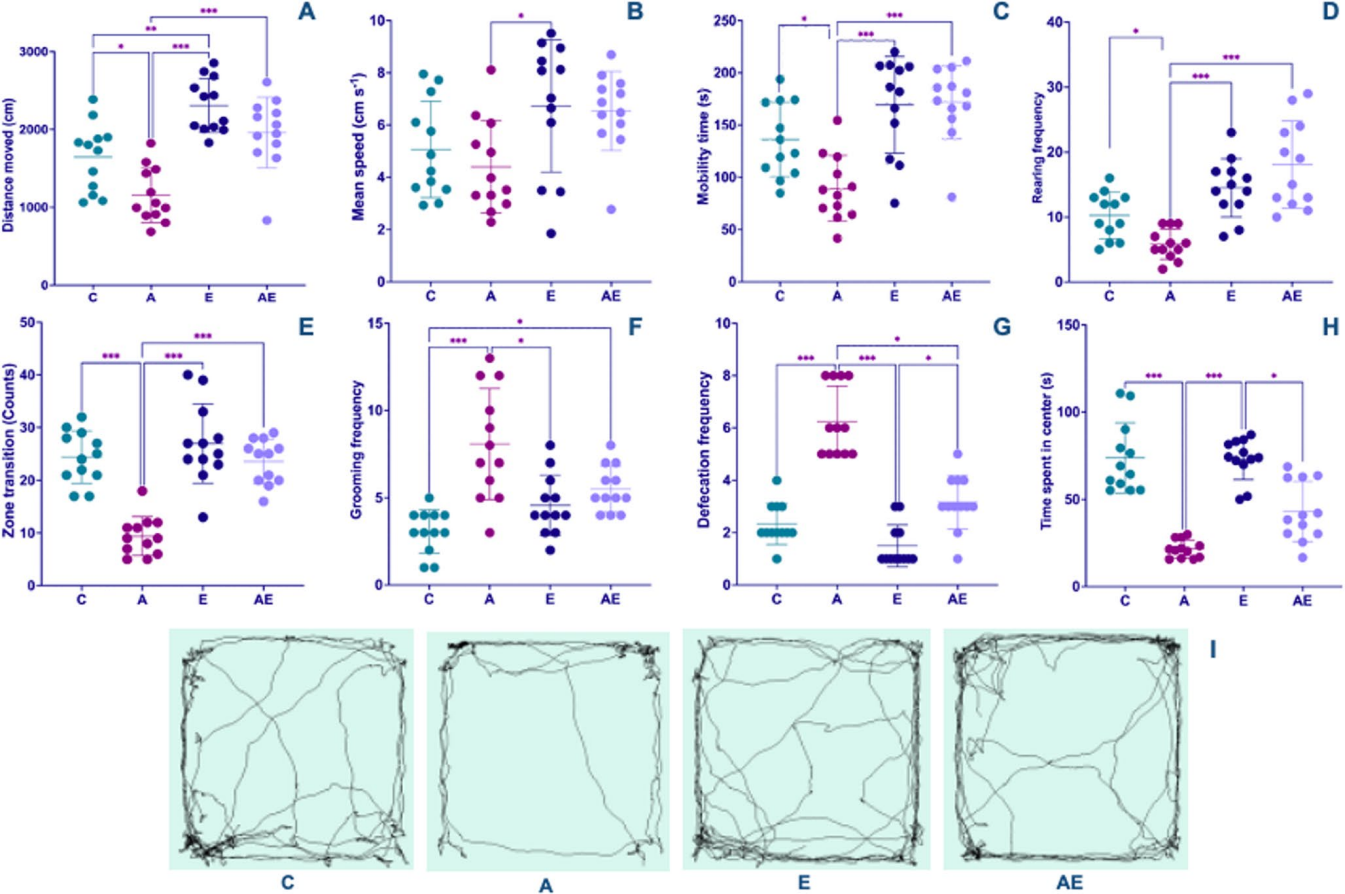
Open field test results of the groups. (**A**) total distance traveled, (**B**) average velocity, (**C**) time spent mobile, reflecting locomotor activity. (**D**) Rearing frequency and (**E**) transitions between peripheral and central zones represent exploratory behavior. (**F**) Grooming frequency, (**G**) number of defecation boli, and (**H**) time spent in the central zone serve as indicators of anxiety-related behavior. (**I**) The track maps of the groups. Data from Control (C), AD (A), Exercise (E), and AD + Exercise (AE) groups (*n* = 12 per group). Data in (A) and (E) were analyzed using a one-way ANOVA followed by Tukey’s HSD test, whereas data in (B), (C), (D), (F), (G), and (H) were analyzed using the non-parametric Kruskal-Wallis test followed by Dunn’s test. Values are depicted as mean ± SD. *p < 0.05, **p < 0.01 and ***p < 0.001

Metrics indicative of overall locomotion within the OF test included total distance moved, average velocity, and duration of active movement. A significant effect was observed for distance traveled across the groups. Post-hoc analysis indicated that the A group covered significantly less total distance than all other experimental cohorts. Conversely, the E group exhibited a higher total distance traversed when compared to the C group (F_(3,44)_ = 18.04, *P* < 0.001) (Fig. 2A and I). Examination of average velocity revealed a significant difference, with the A group demonstrating a lower average speed compared to the E group (Kruskal-Wallis [KW] = 10.05, *P* = 0.02) (Fig. 2B). Furthermore, the A group spent significantly less time actively moving during the OF test relative to all other investigated groups (KW = 20.68, *P* < 0.001) (Fig. 2C).

Exploratory behaviors were assessed by analyzing rearing frequency and the number of transitions between the peripheral and central zones. Significant reductions were observed in both the frequency of rearing events (KW = 26.86, *P* < 0.001) (Fig. 2D) and the number of transitions between peripheral and central areas (F_(3,44)_ = 26.87, *P* < 0.001) (Fig. 2E) for the A group when compared to all other groups.

#### Anxiety-like behavior

Measures of anxiety-related behavior within the OF included grooming frequency, number of defecation boli, and time spent in the central zone. The frequency of grooming, typically associated with increased stress or anxiety, was significantly higher in the A group compared to both the C and E groups. Moreover, the AE group also exhibited higher grooming frequency relative to the C group (KW = 22.70, *P* < 0.001) (Fig. 2F). The number of defecation boli, another somatic indicator of anxiety, was markedly higher in the A group compared to all other experimental groups. Additionally, the AE group displayed a higher number of defecation events when contrasted with the E group (KW = 34.58, *P* < 0.001) (Fig. 2G). Conversely, the time spent in the central zone, a robust inverse correlate of anxiety, was significantly diminished in the A group when compared to both the C and E groups. A significant reduction in central zone occupancy was also evident in the AE group in comparison to the E group (KW = 33.40, *P* < 0.001) (Fig. 2H).

#### Learning and memory function assessments

**Fig. 3:**
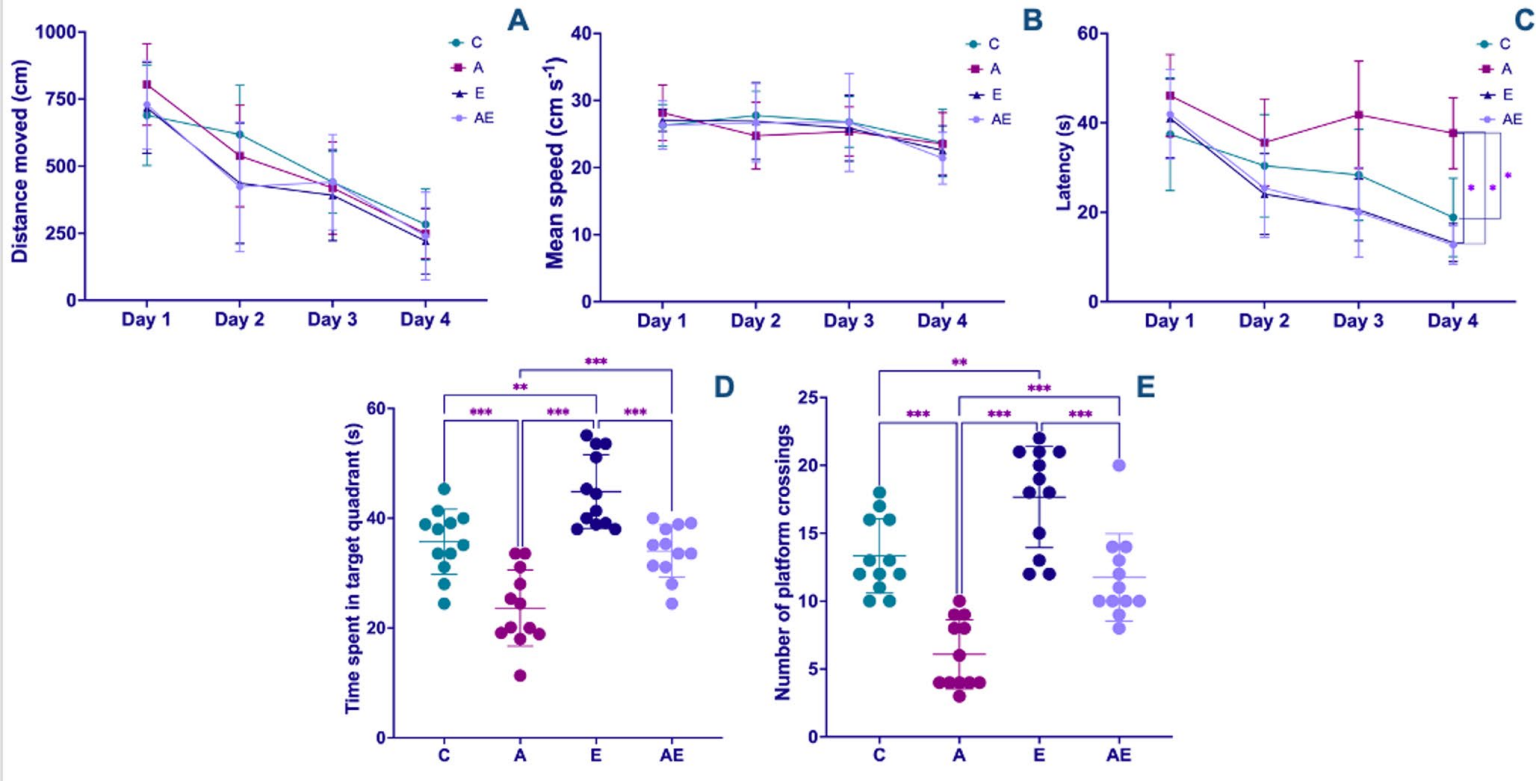
Morris water maze test results. MWM acquisition phase data showing (**A**) total distance traveled, (**B**) average swimming speed, and (**C**) escape latency across training days. During the probe test (**D**) time spent in the target quadrant and (**E**) number of platform crossings. Data from Control (C), AD (A), Exercise (E), and AD + Exercise (AE) groups (*n* = 12 per group). Data were analyzed using a one-way ANOVA followed by Tukey’s HSD test. Values are depicted as mean ± SD. *p < 0.05, **p < 0.01 and ***p < 0.001

A significant effect of repeated measures was observed for total distance traveled, which demonstrated a general reduction across training days (F = 82.43, *P* < 0.001). However, no significant inter-group differences were detected for distance traveled across all measurement time points (F_(3,43)_ = 1.910, *P* = 0.142) (Fig. 3A). Similarly, escape latency exhibited a significant time-dependent decrease, reflecting successful learning across days (F = 57.10, *P* < 0.001). Crucially, the A group displayed significantly prolonged escape latencies when compared to all other experimental groups (F_(3,43)_ = 19.384, *P* < 0.001) (Fig. 3C). For average swimming speed, a significant reduction was observed across repeated measures (F = 9.958, *P* < 0.001), yet no significant distinctions among groups were found at any measurement time (F_(3,43)_ = 0.230, *P* = 0.875) (Fig. 3B).

The MWM probe test, conducted on Day 5 following the four-day acquisition phase, provided insights into long-term spatial memory retention. Analysis revealed that both the time spent in the target quadrant (F_(3,44)_ = 25.84, *P* < 0.001) (Fig. 3D) and the number of crossings over the platform zone (F_(3,44)_ = 28.79, *P* < 0.001) (Fig. 3E) were significantly lower in the A group compared to all other experimental cohorts. Conversely, the E group exhibited significantly higher values for both these memory-related parameters when contrasted with both the C and AE groups.

### Biochemical and molecular analyses

#### Alzheimer’s disease markers and neuroinflammation indicators

**Fig. 4:**
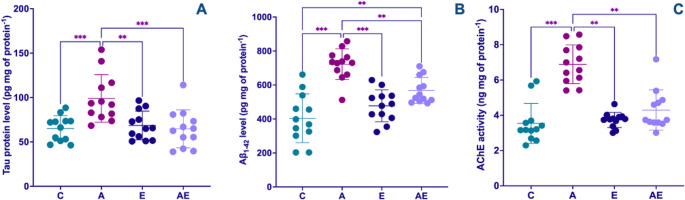
Levels of (A) tau protein, (B) Aβ_1–42_, and (C) AChE activity in hippocampal tissue. Data from Control (C), AD (A), Exercise (E), and AD + Exercise (AE) groups (*n* = 12 per group). Data in (A) and (B) were analyzed using a one-way ANOVA followed by Tukey’s HSD test, whereas data in (C) were analyzed using the non-parametric Kruskal-Wallis test followed by Dunn’s test. Values are depicted as mean ± SD. **p < 0.01 and ***p < 0.001

To characterize hallmark pathological and neuroinflammatory aspects of AD within the hippocampus, specific biomarkers were quantified: Aβ_1−42_ levels, tau protein levels, and AChE activity. Notably, hippocampal tau protein levels were significantly higher in the A group when compared to all other experimental groups (F_(3,44)_ = 7.99, *P* < 0.001) (Fig. 4A). Regarding Aβ_1−42_ accumulation, a significant effect was observed. Post-hoc analysis indicated that the A group exhibited substantially higher Aβ_1–42_ levels compared to all other groups. Furthermore, the AE group demonstrated higher Aβ_1–42_ levels relative to the C group (F_(3,44)_ = 20.81, *P* < 0.001) (Fig. 4B). AChE activity, a marker often dysregulated in neuroinflammatory conditions and AD, was found to be significantly higher in the A group across all comparisons (KW = 26.95, *P* < 0.001) (Fig. 4C).

#### Hippocampal gene expression analysis

**Fig. 5:**
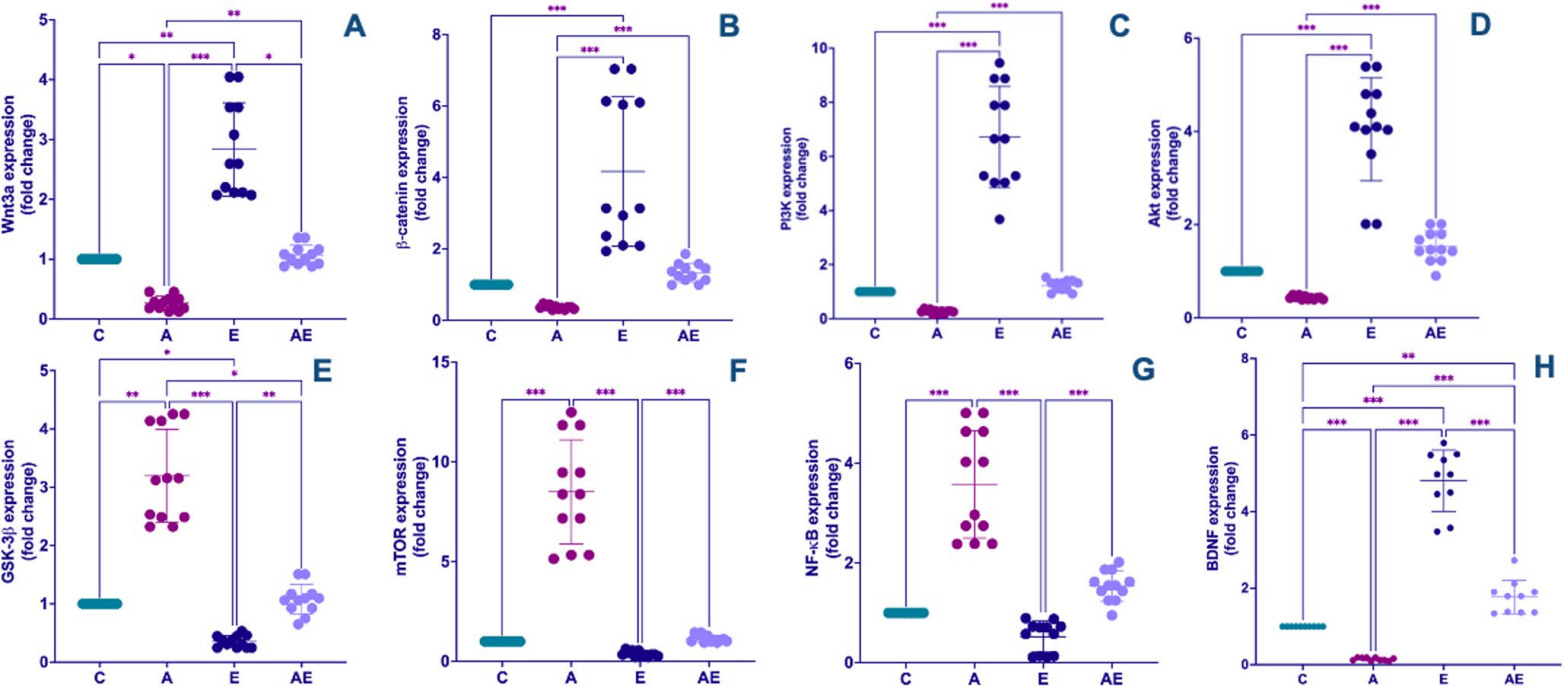
Effects of regular exercise on hippocampal gene expression in an experimental AD model. Gene targets investigated include (**A**) Wnt3a, (**B**) β-catenin, (**C**) PI3K, (**D**) Akt, (**E**) GSK-3β, (**F**) mTOR, (**G**) NF-κB, and (**H**) BDNF. The four experimental groups are: Control (C), AD (A), Exercise (E), and AD + Exercise (AE) (*n* = 12 per group). Data were analyzed using the non-parametric Kruskal-Wallis test followed by Dunn’s test. Data are presented as mean ± SD. Statistical significance levels are indicated by asterisks: * *P* < 0.05, ** *P* < 0.01, and *** *P* < 0.001

Figure 5 illustrates the effects of experimental AD induction and/or exercise training on the hippocampal expression of genes involved in neuroinflammation (NF-κB, mTOR), synaptic plasticity and neuroprotection (Wnt3a, β-catenin, PI3K, Akt), and AD pathology (GSK-3β). Hippocampal Wnt3a mRNA expression was lower in the A group compared to all other groups. Conversely, Wnt3a expression was notably higher in the E group relative to all other groups (KW = 40.83, *P* < 0.001) (Fig. 5A). Correspondingly, β-catenin mRNA level was higher in the E group compared to both the C and A groups. Furthermore, the AE group also demonstrated higher β-catenin expression than the A group (KW = 42.32, *P* < 0.001) (Fig. 5B). Expressions of PI3K (KW = 42.32, *P* < 0.001) (Fig. 5C) and Akt (KW = 43.20, *P* < 0.001) (Fig. 5D) were found to be higher in the E group in comparison to the C and A groups. Moreover, the AE group showed higher levels of PI3K and Akt expressions than the A group. Conversely, GSK-3β mRNA levels were higher in the A group relative to all other groups. However, GSK-3β expression was lower in the E group compared to all other groups (KW = 40.85, *P* < 0.001) (Fig. 5E). mTOR mRNA expression showed a pattern of being significantly higher in the A group compared to both C and E groups, and further elevated in the AE group relative to the E group (KW = 42.32, *P* < 0.001) (Fig. 5F). NF-κB mRNA expression, a key pro-inflammatory mediator, was found to be significantly higher in the A group when compared to the C and E groups, and also higher in the AE group relative to the E group (KW = 43.44, *P* < 0.001) (Fig. 5G). Regarding neurotrophic factors, BDNF mRNA expression was markedly lower in the A group relative to all other groups. In contrast, the E group exhibited the highest BDNF expression, and the AE group also showed significantly higher expression than the C group (KW = 44.80, *P* < 0.001) (Fig. 5H).

#### Changes in GPLD1 expression levels

**Fig. 6:**
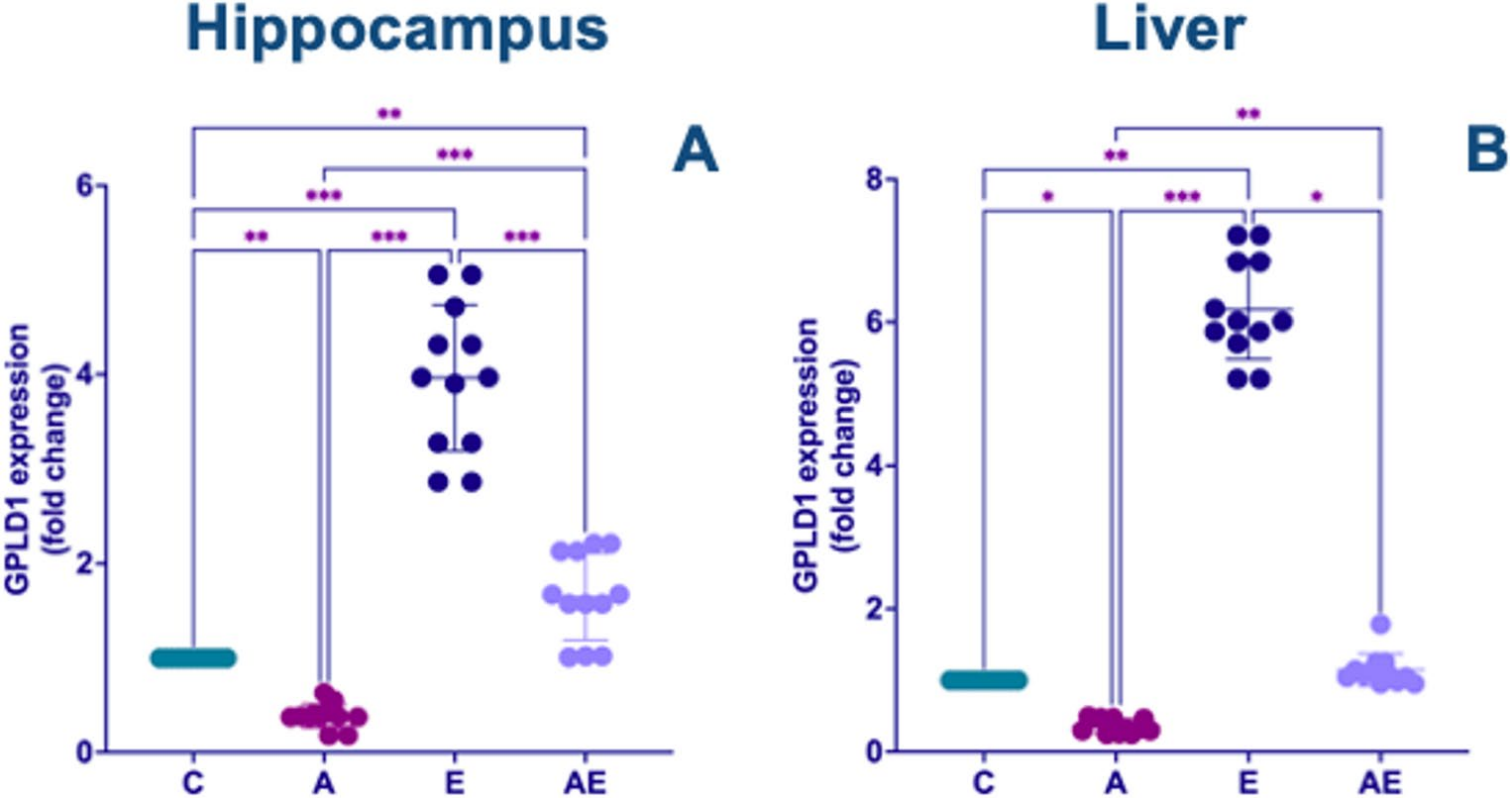
(**A**) Hippocampal and (**B**) liver tissue GPLD1 mRNA expression levels following experimental AD induction and exercise intervention. Data from Control (**C**), AD (A), Exercise (E), and AD + Exercise (AE) groups (*n* = 12 per group). Data in (A) were analyzed using a one-way ANOVA followed by Tukey’s HSD test, whereas data in (B) were analyzed using the non-parametric Kruskal-Wallis test followed by Dunn’s test. Values are depicted as mean ± SD. Statistical significance levels are indicated by asterisks: * *P* < 0.05, ** *P* < 0.01, and *** *P* < 0.001

In the hippocampus, GPLD1 mRNA expression was significantly attenuated in the A group compared to all other experimental groups. Conversely, regular exercise demonstrably upregulated GPLD1 expression, with the E group exhibiting higher levels than all other groups. Furthermore, the AE group showed significantly higher GPLD1 expression relative to the C group (F_(3,44)_ = 143.0, *P* < 0.001) (Fig. 6A). Analysis of the liver tissue revealed a similar pattern for GPLD1 expression. The A group exhibited significantly lower GPLD1 expression levels compared to all other groups. In contrast, the E group displayed markedly higher GPLD1 expression compared to all other groups (KW = 41.45, *P* < 0.001) (Fig. 6B).

#### Changes in GPLD1 and BDNF levels

**Fig. 7:**
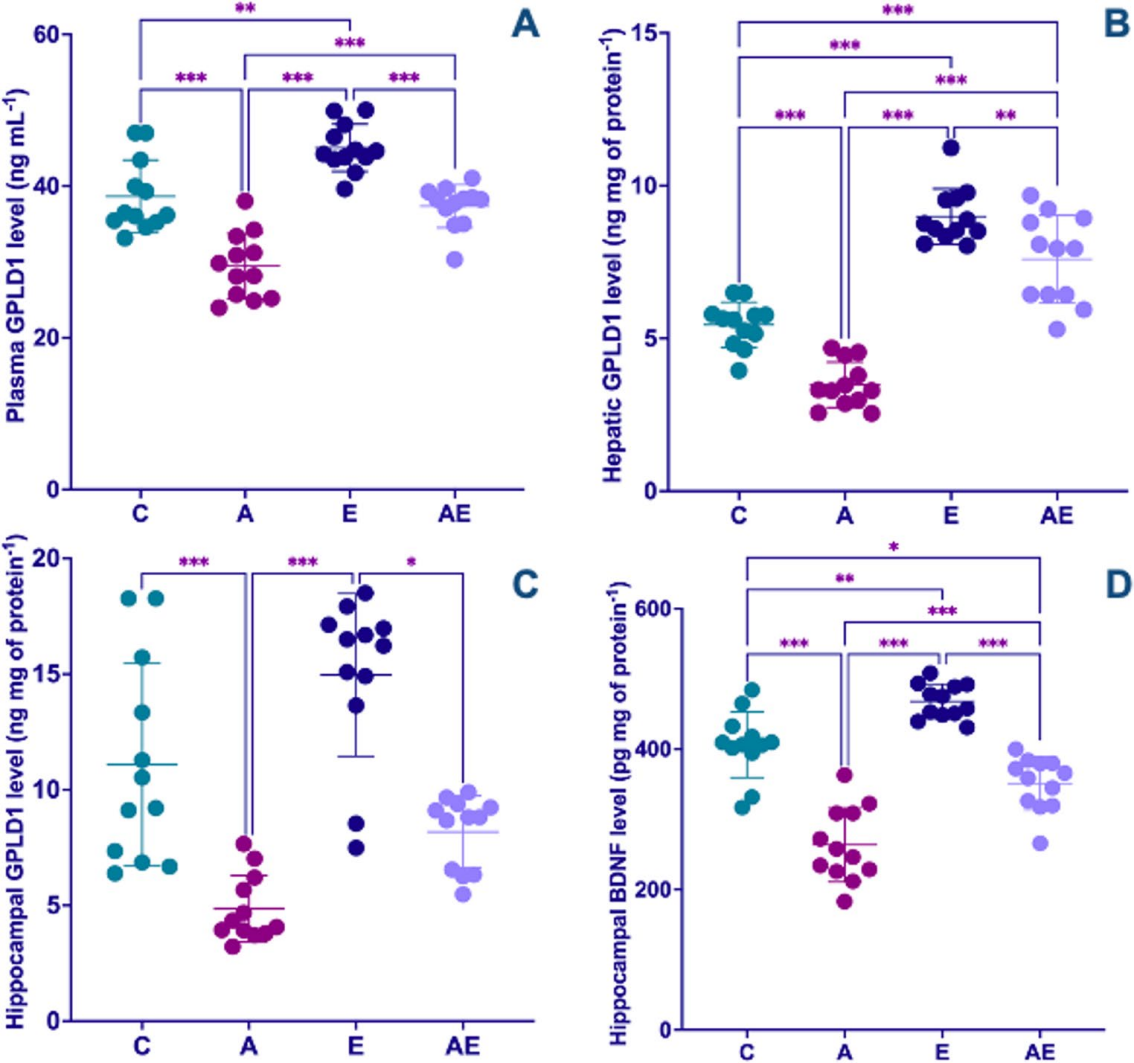
Modulation of quantitative levels of GPLD1 in (**A**) plasma, (**B**) liver, and (**C**) hippocampus, along with (**D**) hippocampal BDNF levels following experimental AD induction and exercise intervention. Data from Control (C), AD (A), Exercise (E), and AD + Exercise (AE) groups (*n* = 12 per group). Data in (A), (B) and (D) were analyzed using a one-way ANOVA followed by Tukey’s HSD test, whereas data in (C) were analyzed using the non-parametric Kruskal-Wallis test followed by Dunn’s test. Values are depicted as mean ± SD. Statistical significance levels are indicated by asterisks: * *P* < 0.05, ** *P* < 0.01, and *** *P* < 0.001

In plasma, GPLD1 level was lower in the A group when compared to all other experimental groups. Conversely, the E group exhibited markedly higher plasma GPLD1 levels than both the C and AE groups (F_(3,44)_ = 33.34, *P* < 0.001) (Fig. 7A). Analysis of liver tissue revealed that GPLD1 level was significantly lower in the A group compared to all other groups. Regular exercise demonstrably elevated liver GPLD1, with the E group showing higher level than the C group. Interestingly, while the AE group showed higher liver GPLD1 levels compared to the C group, these levels were found to be lower than those observed in the E group (F_(3,44)_ = 70.29, *P* < 0.001) (Fig. 7B). Regarding hippocampal GPLD1 level, the A group exhibited significantly lower levels when compared to the C and E groups. Furthermore, the AE group showed lower hippocampal GPLD1 level than the E group (KW = 29.68, *P* < 0.001) (Fig. 7C). In the hippocampus, BDNF level was significantly diminished in the A group relative to all other experimental groups. In contrast, the E group displayed markedly higher hippocampal BDNF level compared to all other groups. The AE group, however, showed lower hippocampal BDNF level compared to the C group (F_(3,44)_ = 51.61, *P* < 0.001) (Fig. 7D).

## Discussion

AD represents a major challenge to public health, characterized by an inexorable neurodegenerative process that dismantles cognitive and functional capacities. The failure of single-target pharmacological agents to halt or reverse this decline has catalyzed a paradigm shift toward multi-target, non-pharmacological interventions, with physical exercise emerging as a uniquely potent strategy for promoting brain resilience (Yang et al. , Brendborg and Febbraio ). However, the fundamental mechanisms by which peripheral muscular activity translates into profound central neuroprotection remain incompletely elucidated. Here, we present a multi-layered investigation that moves beyond the established benefits of exercise to explore a novel mechanistic axis. Our study, employing a validated D-galactose and AlCl_3_-induced rodent model of AD, provides supportive evidence suggesting that regular aerobic exercise may not only ameliorates core pathological and behavioral deficits but potentially does so via a coordinated molecular reprogramming of the hippocampus. Critically, our data implicate the liver-derived exerkine, GPLD1, as a key systemic factor associated with this neuroprotective cascade, thereby suggesting a functional liver-brain interaction that could be explored for future AD therapeutics.

Our initial objective was to rigorously validate our experimental AD model to ensure its fidelity in recapitulating the multifaceted clinical and pathological features of human SAD. The behavioral assessments consistently indicated a phenotype of severe functional decline. We observed substantial deficits in spontaneous locomotor activity and exploratory behavior, characterized by a significant reduction in total distance traveled, average velocity, and total time spent mobile in the OF test. This manifestation of hypokinesia and apathy is not merely a motor artifact but is consistent with findings in other AD models and human patients, which may reflect underlying disruptions in dopaminergic and cholinergic pathways within cortico-striatal-limbic circuits that govern motivation and exploration (Zhang et al. 2024, Snowden et al. 2019, Hsu et al. 2021, Amalric et al. 2021). The anxiogenic profile of the sedentary AD rats, evidenced by a significant increase in stress-induced behaviors such as self-grooming and defecation, coupled with a marked avoidance of the anxiogenic central area of the OF, further highlights the emotional dysregulation central to the AD syndrome. This anxiety phenotype has been proposed in the literature to stem from the hyperactivation of stress circuits and compromised function in limbic structures, particularly the hippocampus and amygdala, which are known to be vulnerable to AD pathology (Pentkowski et al. 2018, Chen et al. 2021).

The most salient behavioral deficit, as anticipated, was the impairment in hippocampus-dependent spatial learning and memory. During the acquisition phase of the MWM, animals in the A group displayed significantly longer escape latencies across all training days, indicative of a severe deficit in spatial learning. Crucially, the absence of a significant difference in swimming speed across groups confirms that this deficit was not due to motor impairment but represents a genuine cognitive failure. This learning impairment culminated in a severe failure of memory consolidation and retrieval, as demonstrated during the probe trial. The sedentary AD rats spent significantly less time in the target quadrant and made fewer crossings over the former platform location, suggesting their inability to recall the learned spatial information (Hoveida et al. 2011, Belviranlı and Okudan 2024). This severe cognitive phenotype provides a notable functional correlate for the biochemical and molecular insults observed in the hippocampus, a brain region known to be one of the earliest and most severely affected by AD pathology (Mufson et al. 2015, Geigenmüller et al. 2024, Li et al. 2025, Ma et al. 2025).

At the molecular level, our AD model effectively modeled key neuropathological hallmarks that define the disease. The significant accumulation of Aβ_1–42_ and elevated total tau protein levels within the hippocampus of the sedentary AD group are consistent with the presence of the core proteinopathies that are widely believed to initiate and drive the synaptic dysfunction, neuroinflammation, and neuronal death characteristic of AD (Long and Holtzman 2019, Selkoe and Hardy 2016). This was further corroborated by the increased activity of AChE, a key enzyme in the cholinergic system. Elevated AChE activity is not merely a consequence of cholinergic neuron loss; it has been increasingly implicated as an active participant in AD pathogenesis by promoting the aggregation of Aβ into toxic plaques (Francis et al. 1999, Moss and Perez 2024). This comprehensive biochemical profile provides a plausible mechanistic basis for the observed cognitive deficits. Concurrent with these pathological changes, our study identified a significant downregulation of endogenous neuroprotective factors. Both the novel candidate GPLD1 and the well-established neurotrophin BDNF were markedly reduced in the hippocampus of AD animals. Given BDNF’s critical role in promoting neuronal survival, synaptogenesis, long-term potentiation, and memory formation, its depletion in the sedentary AD rats represents a potential contributing factor to the observed cognitive decline (Numakawa et al. 2018, Mori et al. 2021). Our novel finding of diminished GPLD1 in the AD context is particularly intriguing, as it suggests that a decline in this systemically-derived factor could be part of the pathological cascade, weakening a potential endogenous defense mechanism against neurodegeneration (Horowitz et al. 2020).

In compelling contrast to this landscape of dysfunction, regular aerobic exercise served as an effective restorative intervention, ameliorating nearly all measured deficits with considerable efficacy. Animals in the healthy exercised group and, critically, the exercised AD groups exhibited marked improvements in behavioral performance compared to their sedentary AD counterparts. The exercise intervention effectively rescued locomotor and exploratory deficits and robustly mitigated anxiety-like behaviors in the AD rats subjected to physical exercise. Most importantly, exercise restored spatial learning and memory function to levels approaching or, in some cases, surpassing that of healthy controls. Exercised AD rats showed significantly improved acquisition curves and a notable rescue of memory in the MWM probe test compared to the sedentary AD group, suggesting that exercise can therapeutically mitigate cognitive impairments even in the face of an ongoing pathological insult. This therapeutic effect aligns with a vast body of literature supporting the potent cognitive benefits of exercise. While our current study primarily focused on specific molecular signaling pathways, previous research has proposed that such behavioral improvements may be driven by broader mechanisms. Based on existing literature, these speculated mechanisms include enhanced adult hippocampal neurogenesis, increased synaptic plasticity, reduced neuroinflammation, and improved cerebral blood flow, which have been frequently reported alongside cognitive assessments in other experimental models (Kivipelto et al. 2018, Praag et al. 1999, Vivar et al. 2013, Valenzuela et al. 2020, Feng et al. 2024, Deng et al. 2025). However, it is important to maintain a critical perspective, as the literature is not entirely uniform. Some clinical and experimental studies have reported limited, highly variable, or even contradictory cognitive outcomes following exercise interventions in AD, suggesting that the neuroprotective efficacy of physical activity may depend heavily on the severity of the disease pathology at the onset of training, the specific exercise modality, and individual metabolic responses.

Our study’s primary objective was to move beyond the phenomenon of exercise’s benefits and explore the underlying molecular mechanisms, with a central hypothesis centered on the role of systemic factors, or “exerkines” (Tari et al. , Townsend et al. 2021, Lu et al. 2025). Inspired by foundational work showing that systemic factors present in young blood can rejuvenate the aged brain and improve cognitive function (Villeda et al. 2011), we focused our investigation on GPLD1. This liver-derived enzyme was recently identified as a potent exerkine capable of reversing age-related cognitive decline in mice (Horowitz et al. 2020). Our results provide supportive, associative evidence for its potential involvement in AD and exercise-associated neuroprotection. As noted, GPLD1 expressions and levels were significantly suppressed by AD induction in both the liver and hippocampus, suggesting its deficiency may contribute to the disease state. Crucially, exercise robustly upregulated GPLD1 mRNA expressions and levels in both the liver and hippocampus of both healthy and AD rats. Additionally, consistent with our findings, it has been reported that physically active older adults exhibit higher plasma GPLD1 level than the sedentary counterparts (Marlatt et al. 2025). This dichotomy—a deficit in disease and a significant increase with a beneficial intervention— suggests a potential link between GPLD1 and a homeostatic, neuroprotective axis (Ansere and Freeman 2020). The significant induction of GPLD1 in the liver reinforces its status as a key hepatokine released into circulation in response to physical activity, potentially acting as a systemic messenger to communicate the benefits of exercise to the brain (Yang et al. 2022). The parallel upregulation of hippocampal BDNF alongside GPLD1 following exercise suggests either a coordinated, independent response to exercise or a potential downstream relationship, although causality cannot be definitively inferred from these observational data. It is plausible that systemic GPLD1 primes or facilitates local BDNF production and signaling within the hippocampus, a hypothesis supported by the findings of Horowitz et al. (Horowitz et al. 2020), who showed that GPLD1 overexpression could increase hippocampal BDNF.

The therapeutic efficacy of exercise in the exercised AD group was mirrored at the level of intracellular signaling pathways, providing a window into the molecular reprogramming of the hippocampus. Exercise intervention appeared to counteract the pathological signaling milieu induced by AD. We observed a significant restoration of neuroprotective and plasticity-related pathways. Exercise upregulated the expression of Wnt3a and its downstream effector β-catenin, which are crucial for synaptic maintenance, dendritic spine stability, and adult neurogenesis—all processes compromised in AD (Jia et al. 2019, Inestrosa et al. 2012). Similarly, exercise reactivated the PI3K/Akt signaling cascade, a central pathway for promoting cell survival, protein synthesis, and neuronal growth, which is known to be suppressed in AD, contributing to apoptosis and atrophy (Um et al. 2011). In concert with the activation of these pro-survival pathways, exercise notably suppressed the activity of pathways implicated in driving AD pathology. GSK-3β, a constitutively active kinase that plays a pivotal, dual role in promoting both Aβ production and the hyperphosphorylation of tau protein, was significantly downregulated in exercised animals, providing a potential mechanism for reducing core AD pathologies (Llorens-Martín et al. 2014). Furthermore, the expression of NF-κB, a master transcriptional regulator of the innate immune response and neuroinflammation, was attenuated by exercise. This suggests that physical activity helps quell the chronic, smoldering inflammatory state that characterizes the AD brain and contributes to a toxic feedback loop of neuronal damage (Koo et al. 2017, Hao et al. 2023, Jiang et al. 2015).

**Fig. 8:**
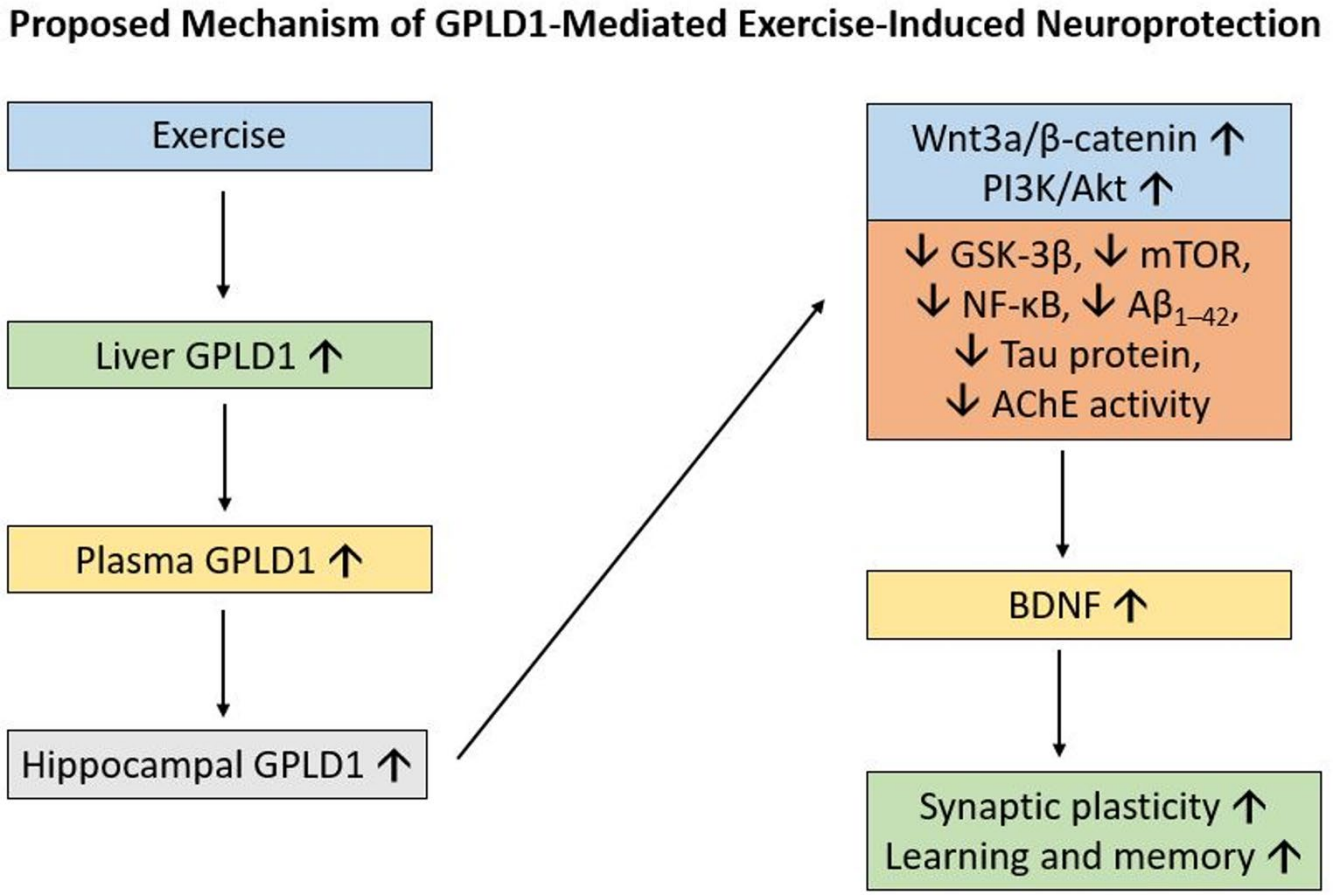
Proposed mechanism of GPLD1-mediated exercise-induced neuroprotection in Alzheimer’s disease

Based on these integrated findings, we propose a plausible mechanistic hypothesis. We postulate that regular physical exercise stimulates the production and release of systemic factors, prominently including the hepatokine GPLD1, into circulation. These systemic signals may potentially initiate a cascade of neuroprotective events within the hippocampus. This likely involves the local upregulation of neurotrophic factors such as BDNF, which then, in concert with other signals, triggers a beneficial reprogramming of intracellular pathways. Specifically, pro-survival pathways like Wnt3a/β-catenin and PI3K/Akt are activated, promoting neuronal resilience and synaptic plasticity. Simultaneously, this environment inhibits the activity of detrimental kinases like GSK-3β and suppresses pro-inflammatory transcription factors like NF-κB. This comprehensive molecular shift creates a virtuous cycle that may actively counteract Aβ and tau pathology, dampen neuroinflammation, support synaptic function, and ultimately contribute to the rescue of cognitive abilities, as evidenced by our consistent behavioral data. While central nervous system processes are inherently complex and multidirectional, this proposed framework offers a potential link between peripheral exercise and central neuronal health, placing GPLD1 as a key upstream correlate in this therapeutic cascade (Fig. 8).

Although the precise mechanism by which circulating GPLD1 influences central hippocampal function was previously ambiguous due to its large size precluding direct passage across the blood-brain barrier (BBB), recent breakthrough evidence provides a compelling vascular explanation. It has been demonstrated that rather than physically crossing the BBB, liver-derived GPLD1 exerts its effects directly on the brain microvasculature. Specifically, GPLD1 enzymatically cleaves a GPI-anchored substrate known as tissue-nonspecific alkaline phosphatase (TNAP) from the luminal surface of brain endothelial cells. AD-related accumulation of cerebrovascular TNAP is known to compromise BBB transport and integrity, leading to inflammation and cognitive decline. By systematically shearing TNAP from the cerebrovasculature, exercise-induced GPLD1 restores BBB integrity, mitigates amyloid-beta pathology, and rescues downstream hippocampal transcriptional signatures (Bieri et al. 2026). Therefore, our observations of exercise-induced systemic GPLD1 upregulation, coupled with substantial central neuroprotection and behavioral recovery, are consistent with this newly established liver-to-brain vascular axis Future targeted studies in our D-galactose/AlCl_3_ model could further validate the specific cerebrovascular alterations mediating this systemic dialogue.

Despite these findings, our study has several limitations. Crucially, the experimental design is associative and lacks causal validation; establishing GPLD1 as a direct mediator would require targeted interventional experiments, such as liver-specific GPLD1 knockdown or antibody neutralization assays. First, the chemically-induced AD model does not fully replicate the complex genetic pathogenesis of human AD, necessitating future validation in transgenic models. Second, we employed a single exercise modality, leaving the “dose-response” effects of different exercise types (e.g., resistance training), intensities, and durations unexplored. Third, the use of male-only animals prevents the assessment of potential sex-based differences in exerkine responses, limiting the generalizability of our findings across biological sexes. Fourth, our study lacked a longitudinal follow-up to determine whether the exercise-induced neuroprotective benefits are sustained after the cessation of the training protocol.

In conclusion, regular physical exercise ameliorates pathological and behavioral deficits in an experimental AD model. This restorative effect is associated with systemic adaptations, notably the upregulation of liver-derived and hippocampal GPLD1, alongside increased BDNF and the favorable reprogramming of key hippocampal signaling pathways. These findings support exercise as an effective non-pharmacological strategy and highlight the GPLD1 signaling axis as a promising candidate for further investigation. Future research exploring such endogenous systemic factors may offer valuable insights into mitigating neurodegeneration and preserving cognitive health.

## Data Availability

No datasets were generated or analysed during the current study.

## References

[CR1] (2023) 2023 Alzheimer’s disease facts and figures. Alzheimers Dement 19(4):1598–1695. 10.1002/alz.13016 10.1002/alz.13016 36918389

[CR47] Alghamdi BSA (2018) Possible prophylactic anti-excitotoxic and anti-oxidant effects of virgin coconut oil on aluminium chloride-induced Alzheimer’s in rat models. J Integr Neurosci 17(3–4):593–607. 10.3233/JIN-180089 30010139 10.3233/JIN-180089

[CR58] Amalric M, Pattij T, Sotiropoulos I, Silva JM, Sousa N, Ztaou S, Chiamulera C, Wahlberg LU, Emerich DF, Paolone G (2021) Where Dopaminergic and Cholinergic Systems Interact: A Gateway for Tuning Neurodegenerative Disorders. Front Behav Neurosci 15:661973. 10.3389/fnbeh.2021.661973 34366802 10.3389/fnbeh.2021.661973 PMC8340002

[CR76] Ansere VA, Freeman WM (2020) Exercising your mind. Science 369(6500):144–145. 10.1126/science.abc8830 32646988 10.1126/science.abc8830

[CR50] Bedford TG, Tipton CM, Wilson NC, Oppliger RA, Gisolfi CV (1979) Maximum oxygen consumption of rats and its changes with various experimental procedures. J Appl Physiol Respir Environ Exerc Physiol 47(6):1278–1283. 10.1152/jappl.1979.47.6.1278 536299 10.1152/jappl.1979.47.6.1278

[CR49] Belviranlı M, Gökbel H, Okudan N, Başaralı K (2012) Effects of grape seed extract supplementation on exercise-induced oxidative stress in rats. Br J Nutr 108(2):249–256. 10.1017/S0007114511005496 22011589 10.1017/S0007114511005496

[CR52] Belviranlı M, Okudan N (2018) Exercise Training Protects Against Aging-Induced Cognitive Dysfunction via Activation of the Hippocampal PGC-1α/FNDC5/BDNF Pathway. Neuromolecular Med 20(3):386–400. 10.1007/s12017-018-8500-3 29971668 10.1007/s12017-018-8500-3

[CR27] Belviranlı M, Okudan N (2019) Voluntary, involuntary and forced exercises almost equally reverse behavioral impairment by regulating hippocampal neurotrophic factors and oxidative stress in experimental Alzheimer’s disease model. Behav Brain Res 364:245–255. 10.1016/j.bbr.2019.02.030 30790584 10.1016/j.bbr.2019.02.030

[CR10] Belviranlı M, Okudan N (2024) Coconut oil ameliorates behavioral and biochemical alterations induced by D-GAL/AlCl3 in rats. Brain Res 1823:148704. 10.1016/j.brainres.2023.148704 38052316 10.1016/j.brainres.2023.148704

[CR51] Belviranlı M, Okudan N, Atalık KE, Öz M (2013) Curcumin improves spatial memory and decreases oxidative damage in aged female rats. Biogerontology 14(2):187–196. 10.1007/s10522-013-9422-y 23609199 10.1007/s10522-013-9422-y

[CR80] Bieri G, Pratt KJB, Fuseya Y, Aghayev T, Sucharov J, Horowitz AM, Philp AR, Fonseca-Valencia K, Chu R, Phan M, Remesal L, Wang SJ, Yang AC, Casaletto KB, Villeda SA (2026) Liver exerkine reverses aging- and Alzheimer’s-related memory loss via vasculature. Cell 189(5):1499–1516e25. 10.1016/j.cell.2026.01.024 41713415 10.1016/j.cell.2026.01.024 PMC13070421

[CR55] Brendborg N, Febbraio MA (2025) Intervention points for the role of physical activity in prevention and treatment of Alzheimer’s disease. J Physiol 16. 10.1113/JP286747 10.1113/JP286747 40237393

[CR60] Chen Y, Dang M, Zhang Z (2021) Brain mechanisms underlying neuropsychiatric symptoms in Alzheimer’s disease: a systematic review of symptom-general and -specific lesion patterns. Mol Neurodegener 16(1):38. 10.1186/s13024-021-00456-1 34099005 10.1186/s13024-021-00456-1 PMC8186099

[CR42] Chiroma SM, Mohd Moklas MA, Mat Taib CN, Baharuldin MTH, Amon Z (2018) d-galactose and aluminium chloride induced rat model with cognitive impairments. Biomed Pharmacother 103:1602–1608. 10.1016/j.biopha.2018.04.152 29864948 10.1016/j.biopha.2018.04.152

[CR25] Cho J, Shin MK, Kim D, Lee I, Kim S, Kang H (2015) Treadmill Running Reverses Cognitive Declines due to Alzheimer Disease. Med Sci Sports Exerc 47(9):1814–1824. 10.1249/MSS.0000000000000612 25574797 10.1249/MSS.0000000000000612

[CR9] Dao AT, Zagaar MA, Salim S, Eriksen JL, Alkadhi KA (2014) Regular exercise prevents non-cognitive disturbances in a rat model of Alzheimer’s disease. Int J Neuropsychopharmacol 17(4):593–602. 10.1017/S1461145713001351 24229510 10.1017/S1461145713001351 PMC4097602

[CR72] Deng T, Yu W, Lü Y (2025) Different physical exercise in the treatment of Alzheimer’s disease. Psychogeriatrics 25(1):e13207. 10.1111/psyg.13207 39460576 10.1111/psyg.13207

[CR12] de Pins B, Cifuentes-Díaz C, Farah AT, López-Molina L, Montalban E, Sancho-Balsells A, López A, Ginés S, Delgado-García JM, Alberch J, Gruart A, Girault JA, Giralt A (2019) Conditional BDNF Delivery from Astrocytes Rescues Memory Deficits, Spine Density, and Synaptic Properties in the 5xFAD Mouse Model of Alzheimer Disease. J Neurosci 39(13):2441–2458. 10.1523/JNEUROSCI.2121-18.2019 30700530 10.1523/JNEUROSCI.2121-18.2019 PMC6435824

[CR20] de Sá Leitão CVF, Moraes BF, Leite GAPD, Duarte AG, da Silva MVG, de Oliveira GM, Andrade FAB, da Silva JAB, Dos Santos RCC, Figueiredo GS, Campos HO, Leite LHR, Drummond LR, Coimbra CC (2025) Twelve weeks of exercise training improves cognitive status, physical performance and quality of life in Alzheimer’s disease: A systematic review and meta-analysis. Ageing Res Rev 104:102655. 10.1016/j.arr.2025.102655 39798804 10.1016/j.arr.2025.102655

[CR21] Dou J, Zhang H, Fu X, Yang Y, Gao X (2025) Optimal dose and type of non-pharmacological treatments to improve cognitive function in people with Alzheimer’s disease: a systematic review and network meta-analysis. Aging Ment Health 29(2):228–237. 10.1080/13607863.2024.2379427 39028199 10.1080/13607863.2024.2379427

[CR46] Duan S, Wang X, Chen G, Quan C, Qu S, Tong J (2018) Inhibiting RIPK1 Limits Neuroinflammation and Alleviates Postoperative Cognitive Impairments in D-Galactose-Induced Aged Mice. Front Behav Neurosci 12:138. 10.3389/fnbeh.2018.00138 30042663 10.3389/fnbeh.2018.00138 PMC6048190

[CR2] Dubois B, Villain N, Frisoni GB, Rabinovici GD, Sabbagh M, Cappa S, Bejanin A, Bombois S, Epelbaum S, Teichmann M, Habert MO, Nordberg A, Blennow K, Galasko D, Stern Y, Rowe CC, Salloway S, Schneider LS, Cummings JL, Feldman HH (2021) Clinical diagnosis of Alzheimer’s disease: recommendations of the International Working Group. Lancet Neurol 20(6):484–496. 10.1016/S1474-4422(21)00066-1 33933186 10.1016/S1474-4422(21)00066-1 PMC8339877

[CR37] Fagerberg L, Hallström BM, Oksvold P, Kampf C, Djureinovic D, Odeberg J, Habuka M, Tahmasebpoor S, Danielsson A, Edlund K, Asplund A, Sjöstedt E, Lundberg E, Szigyarto CA, Skogs M, Takanen JO, Berling H, Tegel H, Mulder J, Nilsson P, Schwenk JM, Lindskog C, Danielsson F, Mardinoglu A, Sivertsson A, von Feilitzen K, Forsberg M, Zwahlen M, Olsson I, Navani S, Huss M, Nielsen J, Ponten F, Uhlén M (2014) Analysis of the human tissue-specific expression by genome-wide integration of transcriptomics and antibody-based proteomics. Mol Cell Proteom 13(2):397–406. 10.1074/mcp.M113.035600 10.1074/mcp.M113.035600 PMC3916642 24309898

[CR71] Feng L, Li B, Yong SS, Wen X, Tian Z (2024) The emerging role of exercise in Alzheimer’s disease: Focus on mitochondrial function. Ageing Res Rev 101:102486. 10.1016/j.arr.2024.102486 39243893 10.1016/j.arr.2024.102486

[CR64] Francis PT, Palmer AM, Snape M, Wilcock GK (1999) The cholinergic hypothesis of Alzheimer’s disease: a review of progress. J Neurol Neurosurg Psychiatry 66(2):137–147. 10.1136/jnnp.66.2.137 10071091 10.1136/jnnp.66.2.137 PMC1736202

[CR7] Geigenmüller JN, Tari AR, Wisloff U, Walker TL (2024) The relationship between adult hippocampal neurogenesis and cognitive impairment in Alzheimer’s disease. Alzheimers Dement 20(10):7369–7383. 10.1002/alz.14179 39166771 10.1002/alz.14179 PMC11485317

[CR23] Hao Z, Liu K, Zhou L, Chen P (2023) Precious but convenient means of prevention and treatment: physiological molecular mechanisms of interaction between exercise and motor factors and Alzheimer’s disease. Front Physiol 14:1193031. 10.3389/fphys.2023.1193031 37362440 10.3389/fphys.2023.1193031 PMC10285460

[CR38] Horowitz AM, Fan X, Bieri G, Smith LK, Sanchez-Diaz CI, Schroer AB, Gontier G, Casaletto KB, Kramer JH, Williams KE, Villeda SA (2020) Blood factors transfer beneficial effects of exercise on neurogenesis and cognition to the aged brain. Science 369(6500):167–173. 10.1126/science.aaw2622 32646997 10.1126/science.aaw2622 PMC7879650

[CR34] Horowitz AM, Villeda SA (2017) Therapeutic potential of systemic brain rejuvenation strategies for neurodegenerative disease. F1000Res 6:1291. 10.12688/f1000research.11437.1 28815019 10.12688/f1000research.11437.1 PMC5539850

[CR8] Hoveida R, Alaei H, Oryan S, Parivar K, Reisi P (2011) Treadmill running improves spatial memory in an animal model of Alzheimer’s disease. Behav Brain Res 216(1):270–274. 10.1016/j.bbr.2010.08.003 20709113 10.1016/j.bbr.2010.08.003

[CR57] Hsu TW, Fuh JL, Wang DW, Chen LF, Chang CJ, Huang WS, Wu HM, Guo WY (2021) Disrupted metabolic connectivity in dopaminergic and cholinergic networks at different stages of dementia from 18F-FDG PET brain persistent homology network. Sci Rep 11(1):5396. 10.1038/s41598-021-84722-8 33686089 10.1038/s41598-021-84722-8 PMC7940645

[CR22] Huuha AM, Norevik CS, Moreira JBN, Kobro-Flatmoen A, Scrimgeour N, Kivipelto M, Van Praag H, Ziaei M, Sando SB, Wisløff U, Tari AR (2022) Can exercise training teach us how to treat Alzheimer’s disease? Ageing Res Rev 75:101559. 10.1016/j.arr.2022.101559 34999248 10.1016/j.arr.2022.101559

[CR78] Inestrosa NC, Montecinos-Oliva C, Fuenzalida M (2012) Wnt signaling: role in Alzheimer disease and schizophrenia. J Neuroimmune Pharmacol 7(4):788–807. 10.1007/s11481-012-9417-5 23160851 10.1007/s11481-012-9417-5

[CR19] Iso-Markku P, Kujala UM, Knittle K, Polet J, Vuoksimaa E, Waller K (2022) Physical activity as a protective factor for dementia and Alzheimer’s disease: systematic review, meta-analysis and quality assessment of cohort and case-control studies. Br J Sports Med 56(12):701–709. 10.1136/bjsports-2021-104981 35301183 10.1136/bjsports-2021-104981 PMC9163715

[CR16] Jia L, Piña-Crespo J, Li Y (2019) Restoring Wnt/β-catenin signaling is a promising therapeutic strategy for Alzheimer’s disease. Mol Brain 12(1):104. 10.1186/s13041-019-0525-5 31801553 10.1186/s13041-019-0525-5 PMC6894260

[CR29] Jiang X, Chai GS, Wang ZH, Hu Y, Li XG, Ma ZW, Wang Q, Wang JZ, Liu GP (2015) CaMKII-dependent dendrite ramification and spine generation promote spatial training-induced memory improvement in a rat model of sporadic Alzheimer’s disease. Neurobiol Aging 36(2):867–876. 10.1016/j.neurobiolaging.2014.10.018 25457025 10.1016/j.neurobiolaging.2014.10.018

[CR41] Jin JK, Jang B, Jin HT, Choi EK, Jung CG, Akatsu H, Kim JI, Carp RI, Kim YS (2015) Phosphatidylinositol-glycan-phospholipase D is involved in neurodegeneration in prion disease. PLoS ONE 10(4):e0122120. 10.1371/journal.pone.0122120 25867459 10.1371/journal.pone.0122120 PMC4395093

[CR5] Kamatham PT, Shukla R, Khatri DK, Vora LK (2024) Pathogenesis, diagnostics, and therapeutics for Alzheimer’s disease: Breaking the memory barrier. Ageing Res Rev 101:102481. 10.1016/j.arr.2024.102481 39236855 10.1016/j.arr.2024.102481

[CR14] Kang EB, Cho JY (2015) Effect of treadmill exercise on PI3K/AKT/mTOR, autophagy, and Tau hyperphosphorylation in the cerebral cortex of NSE/htau23 transgenic mice. J Exerc Nutr Biochem 19(3):199–209. 10.5717/jenb.2015.15090806 10.5717/jenb.2015.15090806 PMC4624121 26527331

[CR33] Katsimpardi L, Litterman NK, Schein PA, Miller CM, Loffredo FS, Wojtkiewicz GR, Chen JW, Lee RT, Wagers AJ, Rubin LL (2014) Vascular and neurogenic rejuvenation of the aging mouse brain by young systemic factors. Science 344(6184):630–634. 10.1126/science.1251141 24797482 10.1126/science.1251141 PMC4123747

[CR30] Ke HC, Huang HJ, Liang KC, Hsieh-Li HM (2011) Selective improvement of cognitive function in adult and aged APP/PS1 transgenic mice by continuous non-shock treadmill exercise. Brain Res 1403:1–11. 10.1016/j.brainres.2011.05.056 21689809 10.1016/j.brainres.2011.05.056

[CR67] Kivipelto M, Mangialasche F, Ngandu T (2018) Lifestyle interventions to prevent cognitive impairment, dementia and Alzheimer disease. Nat Rev Neurol 14(11):653–666. 10.1038/s41582-018-0070-3 30291317 10.1038/s41582-018-0070-3

[CR15] Koo JH, Kang EB, Oh YS, Yang DS, Cho JY (2017) Treadmill exercise decreases amyloid-β burden possibly via activation of SIRT-1 signaling in a mouse model of Alzheimer’s disease. Exp Neurol 288:142–152. 10.1016/j.expneurol.2016.11.014 27889467 10.1016/j.expneurol.2016.11.014

[CR43] LaFerla FM, Green KN (2012) Animal models of Alzheimer disease. Cold Spring Harb Perspect Med 2(11):a006320. 10.1101/cshperspect.a006320 23002015 10.1101/cshperspect.a006320 PMC3543097

[CR44] León-Arcia K, Andrade-Guerrero J, Martínez-Orozco H, Villegas-Rojas MM, Pérez-Segura I, Ramírez IL, Vilches-Flores A, Guerra-Crespo M, Díaz-Miranda SY, Soto-Rojas LO (2026) First unified time-course of Alzheimer’s-like pathology in the intracerebroventricular streptozotocin-rat model: A systematic review. Ageing Res Rev 113:102918. 10.1016/j.arr.2025.102918 41093165 10.1016/j.arr.2025.102918

[CR61] Li H, Zhao Z, Fassini A, Lee HK, Green RJ, Gomperts SN (2025) Impaired hippocampal circuit function underlying memory encoding and consolidation precede robust Aβ deposition in a mouse model of alzheimer’s disease. Sci Rep 15(1):21957. 10.1038/s41598-025-05653-2 40594747 10.1038/s41598-025-05653-2 PMC12217341

[CR17] Llorens-Martín M, Jurado J, Hernández F, Avila J (2014) GSK-3β, a pivotal kinase in Alzheimer disease. Front Mol Neurosci 7:46. 10.3389/fnmol.2014.00046 24904272 10.3389/fnmol.2014.00046 PMC4033045

[CR3] Long JM, Holtzman DM (2019) Alzheimer Disease: An Update on Pathobiology and Treatment Strategies. Cell 179(2):312–339. 10.1016/j.cell.2019.09.001 31564456 10.1016/j.cell.2019.09.001 PMC6778042

[CR75] Lu X, Chen Y, Shi Y, Shi Y, Su X, Chen P, Wu D, Shi H (2025) Exercise and exerkines: Mechanisms and roles in anti-aging and disease prevention. Exp Gerontol 200:112685. 10.1016/j.exger.2025.112685 39818278 10.1016/j.exger.2025.112685

[CR24] Lu Y, Dong Y, Tucker D, Wang R, Ahmed ME, Brann D, Zhang Q (2017) Treadmill Exercise Exerts Neuroprotection and Regulates Microglial Polarization and Oxidative Stress in a Streptozotocin-Induced Rat Model of Sporadic Alzheimer’s Disease. J Alzheimers Dis 56(4):1469–1484. 10.3233/JAD-160869 28157094 10.3233/JAD-160869 PMC5450951

[CR26] Maliszewska-Cyna E, Xhima K, Aubert I (2016) A Comparative Study Evaluating the Impact of Physical Exercise on Disease Progression in a Mouse Model of Alzheimer’s Disease. J Alzheimers Dis 53(1):243–257. 10.3233/JAD-150660 27163797 10.3233/JAD-150660

[CR62] Ma L, Wei Q, Jiang M, Wu Y, Liu X, Yang Q, Bai Z, Yang L (2025) Hippocampal Neurogenesis in Alzheimer’s Disease: Multimodal Therapeutics and the Neurogenic Impairment Index Framework. Int J Mol Sci 26(13):6105. 10.3390/ijms26136105 40649882 10.3390/ijms26136105 PMC12250571

[CR36] Mann KJ, Hepworth MR, Raikwar NS, Deeg MA, Sevlever D (2004) Effect of glycosylphosphatidylinositol (GPI)-phospholipase D overexpression on GPI metabolism. Biochem J 378(Pt 2):641–648. 10.1042/BJ20031326 14611645 10.1042/BJ20031326 PMC1223959

[CR39] Marlatt KL, Lowe AC, Sanchez-Delgado G, Beyl RA, Viverito MK, Keller JN, Carmichael OT, Ravussin E (2025) Associations between physical activity, brain health, cognitive function, and circulating GPLD1 in healthy older (65–85 years) individuals. Geroscience 47(3):3821–3834. 10.1007/s11357-024-01459-8 39762691 10.1007/s11357-024-01459-8 PMC12181451

[CR13] Mitroshina EV, Yarkov RS, Mishchenko TA, Krut’ VG, Gavrish MS, Epifanova EA, Babaev AA, Vedunova MV (2020) Brain-Derived Neurotrophic Factor (BDNF) Preserves the Functional Integrity of Neural Networks in the β-Amyloidopathy Model in vitro. Front Cell Dev Biol 8:582. 10.3389/fcell.2020.00582 32733889 10.3389/fcell.2020.00582 PMC7360686

[CR28] Moore KM, Girens RE, Larson SK, Jones MR, Restivo JL, Holtzman DM, Cirrito JR, Yuede CM, Zimmerman SD, Timson BF (2016) A spectrum of exercise training reduces soluble Aβ in a dose-dependent manner in a mouse model of Alzheimer’s disease. Neurobiol Dis 85:218–224. 10.1016/j.nbd.2015.11.004 26563933 10.1016/j.nbd.2015.11.004

[CR66] Mori Y, Tsuji M, Oguchi T, Kasuga K, Kimura A, Futamura A, Sugimoto A, Kasai H, Kuroda T, Yano S, Hieda S, Kiuchi Y, Ikeuchi T, Ono K (2021) Serum BDNF as a Potential Biomarker of Alzheimer’s Disease: Verification Through Assessment of Serum, Cerebrospinal Fluid, and Medial Temporal Lobe Atrophy. Front Neurol 12:653267. 10.3389/fneur.2021.653267 33967943 10.3389/fneur.2021.653267 PMC8102980

[CR65] Moss DE, Perez RG (2024) The phospho-tau cascade, basal forebrain neurodegeneration, and dementia in Alzheimer’s disease: Anti-neurodegenerative benefits of acetylcholinesterase inhibitors. J Alzheimers Dis 102(3):617–626. 10.1177/13872877241289602 39533696 10.1177/13872877241289602

[CR6] Mufson EJ, Mahady L, Waters D, Counts SE, Perez SE, DeKosky ST, Ginsberg SD, Ikonomovic MD, Scheff SW, Binder LI (2015) Hippocampal plasticity during the progression of Alzheimer’s disease. Neuroscience 309:51–67. 10.1016/j.neuroscience.2015.03.006 25772787 10.1016/j.neuroscience.2015.03.006 PMC4567973

[CR31] Nigam SM, Xu S, Kritikou JS, Marosi K, Brodin L, Mattson MP (2017) Exercise and BDNF reduce Aβ production by enhancing α-secretase processing of APP. J Neurochem 142(2):286–296. 10.1111/jnc.14034 28382744 10.1111/jnc.14034 PMC5498234

[CR11] Numakawa T, Odaka H, Adachi N (2018) Actions of Brain-Derived Neurotrophin Factor in the Neurogenesis and Neuronal Function, and Its Involvement in the Pathophysiology of Brain Diseases. Int J Mol Sci 19(11):3650. 10.3390/ijms19113650 30463271 10.3390/ijms19113650 PMC6274766

[CR59] Pentkowski NS, Berkowitz LE, Thompson SM, Drake EN, Olguin CR, Clark BJ (2018) Anxiety-like behavior as an early endophenotype in the TgF344-AD rat model of Alzheimer’s disease. Neurobiol Aging 61:169–176. 10.1016/j.neurobiolaging.2017.09.024 29107184 10.1016/j.neurobiolaging.2017.09.024 PMC7944488

[CR35] Raymond FD, Fortunato G, Moss DW, Castaldo G, Salvatore F, Impallomeni M (1994) Inositol-specific phospholipase D activity in health and disease. Clin Sci (Lond) 86(4):447–451. 10.1042/cs0860447 8168340 10.1042/cs0860447

[CR53] Schmittgen TD, Livak KJ (2008) Analyzing real-time PCR data by the comparative C(T) method. Nat Protoc 3(6):1101–1108. 10.1038/nprot.2008.73 18546601 10.1038/nprot.2008.73

[CR40] Seki T, Kanagawa M, Kobayashi K, Kowa H, Yahata N, Maruyama K, Iwata N, Inoue H, Toda T (2020) Galectin 3-binding protein suppresses amyloid-β production by modulating β-cleavage of amyloid precursor protein. J Biol Chem 295(11):3678–3691. 10.1074/jbc.RA119.008703 31996371 10.1074/jbc.RA119.008703 PMC7076203

[CR63] Selkoe DJ, Hardy J (2016) The amyloid hypothesis of Alzheimer’s disease at 25 years. EMBO Mol Med 8(6):595–608. 10.15252/emmm.201606210 27025652 10.15252/emmm.201606210 PMC4888851

[CR56] Snowden SG, Ebshiana AA, Hye A, Pletnikova O, O’Brien R, Yang A, Troncoso J, Legido-Quigley C, Thambisetty M (2019) Neurotransmitter Imbalance in the Brain and Alzheimer’s Disease Pathology. J Alzheimers Dis 72(1):35–43. 10.3233/JAD-190577 31561368 10.3233/JAD-190577 PMC12961646

[CR18] Sujkowski A, Hong L, Wessells RJ, Todi SV (2022) The protective role of exercise against age-related neurodegeneration. Ageing Res Rev 74:101543. 10.1016/j.arr.2021.101543 34923167 10.1016/j.arr.2021.101543 PMC8761166

[CR73] Tari AR, Norevik CS, Scrimgeour NR, Kobro-Flatmoen A, Storm-Mathisen J, Bergersen LH, Wrann CD, Selbæk G, Kivipelto M, Moreira JBN, Wisløff U (2019) Are the neuroprotective effects of exercise training systemically mediated? Prog Cardiovasc Dis 62(2):94–101. 10.1016/j.pcad.2019.02.003 30802460 10.1016/j.pcad.2019.02.003

[CR74] Townsend LK, MacPherson REK, Wright DC (2021) New Horizon: Exercise and a Focus on Tissue-Brain Crosstalk. J Clin Endocrinol Metab 106(8):2147–2163. 10.1210/clinem/dgab333 33982072 10.1210/clinem/dgab333

[CR79] Um HS, Kang EB, Koo JH, Kim HT, Jin-Lee, Kim EJ, Yang CH, An GY, Cho IH, Cho JY (2011) Treadmill exercise represses neuronal cell death in an aged transgenic mouse model of Alzheimer’s disease. Neurosci Res 69(2):161–173. 10.1016/j.neures.2010.10.004 20969897 10.1016/j.neures.2010.10.004

[CR70] Valenzuela PL, Castillo-García A, Morales JS, de la Villa P, Hampel H, Emanuele E, Lista S, Lucia A (2020) Exercise benefits on Alzheimer’s disease: State-of-the-science. Ageing Res Rev 62:101108. 10.1016/j.arr.2020.101108 32561386 10.1016/j.arr.2020.101108

[CR45] Van Dam D, De Deyn PP (2006) Drug discovery in dementia: the role of rodent models. Nat Rev Drug Discov 5(11):956–970. 10.1038/nrd2075 17080031 10.1038/nrd2075

[CR68] van Praag H, Christie BR, Sejnowski TJ, Gage FH (1999) Running enhances neurogenesis, learning, and long-term potentiation in mice. Proc Natl Acad Sci USA 96(23):13427–13431. 10.1073/pnas.96.23.13427 10557337 10.1073/pnas.96.23.13427 PMC23964

[CR32] Villeda SA, Luo J, Mosher KI, Zou B, Britschgi M, Bieri G, Stan TM, Fainberg N, Ding Z, Eggel A, Lucin KM, Czirr E, Park JS, Couillard-Després S, Aigner L, Li G, Peskind ER, Kaye JA, Quinn JF, Galasko DR, Xie XS, Rando TA, Wyss-Coray T (2011) The ageing systemic milieu negatively regulates neurogenesis and cognitive function. Nature 477(7362):90–94. 10.1038/nature10357 21886162 10.1038/nature10357 PMC3170097

[CR69] Vivar C, Potter MC, van Praag H (2013) All about running: synaptic plasticity, growth factors and adult hippocampal neurogenesis. Curr Top Behav Neurosci 15:189–210. 10.1007/7854_2012_220 22847651 10.1007/7854_2012_220 PMC4565722

[CR48] Xiao F, Li XG, Zhang XY, Hou JD, Lin LF, Gao Q, Luo HM (2011) Combined administration of D-galactose and aluminium induces Alzheimer-like lesions in brain. Neurosci Bull 27(3):143–155. 10.1007/s12264-011-1028-2 21614097 10.1007/s12264-011-1028-2 PMC5560362

[CR77] Yang AC, Vest RT, Kern F, Lee DP, Agam M, Maat CA, Losada PM, Chen MB, Schaum N, Khoury N, Toland A, Calcuttawala K, Shin H, Pálovics R, Shin A, Wang EY, Luo J, Gate D, Schulz-Schaeffer WJ, Chu P, Siegenthaler JA, McNerney MW, Keller A, Wyss-Coray T (2022) A human brain vascular atlas reveals diverse mediators of Alzheimer’s risk. Nature 603(7903):885–892. 10.1038/s41586-021-04369-3 35165441 10.1038/s41586-021-04369-3 PMC9635042

[CR54] Yang D, Hou N, Jia M (2025) Multicomponent exercise interventions for older adults with Alzheimer’s disease: a systematic review and meta-analytical perspective. J Alzheimers Dis 13872877251346989. 10.1177/13872877251346989 10.1177/13872877251346989 40452360

[CR4] Zhang J, Zhang Y, Wang J, Xia Y, Zhang J, Chen L (2024) Recent advances in Alzheimer’s disease: Mechanisms, clinical trials and new drug development strategies. Signal Transduct Target Ther 9(1):211. 10.1038/s41392-024-01911-3 39174535 10.1038/s41392-024-01911-3 PMC11344989

